# In-kind incentives and health worker performance: Experimental evidence from El Salvador

**DOI:** 10.1016/j.jhealeco.2019.102267

**Published:** 2020-03

**Authors:** Pedro Bernal, Sebastian Martinez

**Affiliations:** Inter-American Development Bank, 1300 New York Avenue, NW, Washington, DC, 20577, United States

**Keywords:** Pay for performance, Performance incentives, In-kind, Team incentives, Health services, El Salvador

## Abstract

We experimentally evaluated the effects of in-kind team incentives on health worker performance in El Salvador, with 38 out of 75 community health teams randomly assigned to performance incentives over a 12-month period. All teams received monitoring, performance feedback and recognition for their achievements allowing us to isolate the effect of the incentive. While both treatment and control groups exhibit improvements in performance measures over time, the in-kind incentives generated significant improvements in community outreach, quality of care, timeliness of care, and utilization of maternal and child health services after 12 months. Gains were largest for teams at the bottom and top of the baseline performance distribution. We find no evidence of results being driven by changes in reporting or by shifting away effort from non-contracted outcomes. These results suggest that in-kind team incentives may be a viable alternative to monetary or individual incentives in certain contexts.

## 1 Introduction

Deficient health provider performance has been documented in multiple middle and low-income countries, including failure to meet coverage targets, absenteeism from post of duty, non-compliance with clinical guidelines and even malpractice (Berendes et al., 2011; Das and Hammer, 2004, 2007; Das et al., 2012; Banerjee et al., 2004; Barber et al., 2007). While the causes of poor performance are complex, provider effort is thought to play an important role as providers often fail to do what is within their knowledge and means. Providers may fail to comply with clinical guidelines even when they have adequate knowledge and resources (Das et al., 2008). As a response, public and private health administrators have implemented incentive schemes that reward providers for accomplishing determined health outputs or outcomes (Miller and Babiarz, 2014; Basinga et al., 2011; Bonfrer et al., 2013; de Walque et al., 2015).

We study the effects of in-kind group incentives for health teams composed of doctors, nurses and community health workers in El Salvador’s public sector. Incentives were based on eleven maternal and child health targets, measured every six months using medical record reviews and household surveys. Health teams were awarded points for each target met, and points were redeemable for in-kind incentives such as computers, microwaves, air-conditioners and other workplace assets to be shared by the team members. The scheme used a non-linear sliding scale, in which teams could claim between $650 and $1000 worth of in-kind incentives after achieving a minimum number of points in each six-month cycle.

We identify the incentive effect using a randomized controlled trial. Thirty-eight out of 75 teams were randomly assigned to receive incentives during the first twelve months of the pilot. The control group became eligible for incentives thereafter. All teams received the same performance feedback, supervision, and recognition for their achievements, allowing us to isolate the effect of the in-kind incentives. Furthermore, the experiment was conducted with existing health teams already operating within the organizational structure of the Ministry of Health, allowing us to overcome potential selection of health workers into the incentive model or selection of individuals into teams.

In-kind incentives led to improved performance on a variety of dimensions, including community outreach, quality of care, timeliness of care, and utilization of services. Average standardized effects ranged from 0.10 to 0.17 standard deviations, with the largest effects on community outreach (0.17 standard deviations) and quality of care (0.14 standard deviations). Substantial gains were achieved by teams with the lowest baseline performance, whose gains centered on activities requiring less team member coordination such as quality of care which is influenced mostly by physicians, and community outreach, which is mostly the responsibility of community health workers. Even larger gains were observed for teams with the highest baseline performance, including for more complex outcomes that require changing patient behaviors such as timeliness of prenatal and postnatal care and utilization of health services including family planning, deworming of children and MMR vaccination. Teams with intermediate performance at baseline that required little effort to meet the performance goals exhibit no response to the incentive scheme. Our results are robust to several specifications and we find no evidence of them being driven by changes in reporting or by shifting effort away from non-contracted outcomes.

Our contribution to the pay-for-performance in health literature is fourfold.^1^ First, pay-for-performance interventions typically bundle incentives with audits, feedback and information, and even public recognition, thus obscuring the effect of incentives alone (Mendelson et al., 2017; Ivers et al., 2012). We conduct one of the first experiments to isolate the effects of incentives from information and public recognition.^2^ Second, we contribute to the literature on team incentives and show that they can be effective particularly in settings were teams are small, members have clearly defined roles and their activities are interdependent. Previous evidence on the effect of team-level incentives has been concentrated primarily on private firms (Friebel et al., 2017; Boning et al., 2007), and less is known for the public and health sectors.

Third, we show that modest in-kind incentives can produce substantial effects on outreach, quality and coverage of public health services. Compared to monetary incentives, in-kind incentives may be less prone to controversy and expectations of permanency as part of remuneration packages. As such, they may be easier to negotiate in settings of strong unions and with administrators hesitant to assume permanent financial commitments. While traditional economic theory suggests that in-kind incentives are no better than an equivalent monetary amount^3^ (Waldfogel, 1996), findings from the behavioral economics and social psychology literature suggest that in some settings in-kind incentives could elicit greater effort (Kube et al., 2012; Heyman and Ariely, 2004) and be less prone to backfiring by crowding out intrinsic motivation (Lacetera and Macis, 2010).^4^ A common explanation for this finding is that monetary incentives might be perceived as a payment in a market interaction, whereas in-kind incentives might be perceived as a gift-giving act that elicits loyalty and a response independent of the gift value (Heyman and Ariely, 2004; Bareket-Bojmel et al., 2017). Moreover, recipients of team in-kind incentives do not only derive utility from the material value of the goods (i.e using a microwave they earned), but also symbolic and social utility from it, as it can be a salient reminder of the team’s performance from which team-members can derive pride (Lacetera et al., 2012; Scott, 2004). To our knowledge, there is no previous evidence of the effect of team-based in-kind incentives in a field experiment.^5^

Finally, we show heterogeneous responses with respect to baseline performance. While the literature discusses some common concerns of non-linear incentive schemes, such as a potential failure to motivate those further away from performance targets (Miller and Babiarz, 2014), there is little empirical evidence on this. Our results suggest that the non-linear scheme implemented in El Salvador improved performance for those well below the incentive threshold as well as for those substantially above this threshold at baseline.

The paper is organized as follows. The next section describes El Salvador’s health system and the Mesoamerica Health Initiative under which the pay for performance experiment took place. The third section describes the incentive scheme, how performance was measured, and feedback was provided. The fourth section discusses data sources and the fifth section presents our identification strategy. Results are presented in section six. In section seven we discuss robustness checks and potential alternative explanations for our results. Section eight discusses the results and concludes.

## 2 Background

### 2.1 The health system in El Salvador^6^

Starting in 2010, El Salvador implemented a public health system reform aimed at improving access to primary care services through the creation of government funded and managed community health teams.^7^ The country’s health reform was implemented by stages, prioritizing the poorest municipalities with a rapid expansion of health units between 2010 and 2013.^8^ Health teams covered the primary care needs of the population in pre-defined geographical catchment areas composed of approximately 3000 individuals in rural areas and 9000 individuals in urban areas. Each rural team was composed of seven members: a physician, a professional nurse, an auxiliary nurse, three community health workers (CHWs, one for every 200 families) and a multi-purpose worker. Urban teams had the same composition but increased the number of CHWs to six (one for every 300 families). Teams had a clearly defined portfolio of approximately 300 primary health care services which included health education and promotion, preventive care, curative care, and community-based rehabilitation.

To provide these services, teams conducted a census of their catchment areas to obtain health and demographic data used to generate a health-risk profile of families and individuals. This risk profile determined the services required by patients according to guidelines (MINSAL, 2011). For instance, for children 24–60 months old with no health risks, CHWs should provide home-visits twice per year and refer to well-visits^9^ with a physician or professional nurse in the health unit twice a year. Children younger than 24 months should receive home-visits and well-visits in the unit every other month up to 12 months of age and every three months up to 24 months of age. The guidelines contained job-descriptions for each member of the team and their role in providing the established services according to the risk profile of the population.

### 2.2 Salud Mesoamerica Initiative

Financing of the health reform combined national and donor funds, including those from the *Salud Mesoamerica Initiative* (SMI). SMI is a public-private partnership^10^ that aimed to reduce maternal and child health inequalities in the Mesoamerica region^11^ by extending coverage and improving the quality of health care for the poor. SMI combined grants equal to 50 percent of the project value with an equivalent percent of country funding. The funding was provided with a Results-Based Aid (RBA) model in which national country governments are offered an incentive equal to 25 percent of the total funding envelope if they achieve 80 percent of pre-established targets at the end of a 24-month funding period (Bernal et al., 2018). The incentive SMI offered to national governments had no restrictions, except being earmarked for the health sector. In the case of El Salvador, SMI provided funding for the country’s health reform in 14 of the poorest municipalities (see Table A1 in the Appendix for the list of municipalities)^12^ through two 24-month operations.^13^ The in-kind incentives for health teams studied here was introduced as part of SMI’s second phase, in October 2015, in an effort to motivate health teams and improve the quality of services.

## 3 Description of the intervention

### 3.1 Incentive scheme

The intervention consisted of an in-kind group incentive to health teams linked to 11 key maternal and child health indicators that mapped closely to SMI’s performance framework at a national level, covering outcomes related to family planning, prenatal, postnatal and child care. The indicators were designed to promote community outreach, increase utilization, and improve the timeliness and quality of care. Each indicator was assigned a performance target. If a community health team met the target, it received points redeemable for in-kind incentives. Targets were set based on SMI’s baseline information (Mokdad et al., 2015), the feasibility of reaching the target over 24-month period, and their alignment to SMI’s performance framework. A description of indicators, targets and their points can be found in Table 1. Points for each indicator were weighted based on the expected level of effort to achieve the target and ranged from 5 to 15 points.

**Table 1:** Indicators used to evaluate team performance, targets, and source of verification.

Category	Indicator	Target	Points	Source
Outreach	Women 15–49 receiving information on modern family planning methods by health personnel in the last six months	80%	5	Household survey
Outreach	Women 15–49 with children less than five with knowledge of treatment of diarrhea with oral rehydration salts and zinc at the time of the survey	50%	10	Household survey
Quality	Prenatal care according to national clinical guidelines for women with a delivery in the last four months	80%	10	Medical Records
Quality	Reference to institutional delivery in birth plan for women with a delivery in the last four months	100%	5	Medical Records
Timeliness	First prenatal care visit prior to 12 weeks of gestation for women with pregnancies that reached three months of gestation in the last six months	80%	10	Medical Records
Timeliness	Postpartum care within a week from delivery by health personnel for women with a delivery in the last four months	92%	10	Medical Records
Utilization	Women 15–49 in need of family planning using a modern family planning method at the time of the survey	61%	15	Household survey
Utilization	Institutional Delivery for women with a delivery in the last four months	94%	10	Medical Records
Utilization	Prescription of micronutrients for children 6–23 months old in the last six months^1/^	80%	15	Medical Records
Utilization	Consumption of two deworming pills in the last six months by children 18–59 months old^2/^	80%	5	Household survey
Utilization	Children 12–23 months with vaccination of measles, mumps, and rubella (MMR)	80%	5	Medical Records
	Total points		100	

*Notes*: The target is the one set by the MoH for the indicator in order to receive the points towards the performance score. The points are the ones awarded to the performance score if the target was met. The source describes the verification source for the target. The detailed definition of each indicator can be found in Table A2 in the Appendix. Prescription of micronutrients is included in the utilization category since the underlying outcome of interest was to improve the consumption of micronutrients sachets, but no feasible indicators could be used other than this.

1/ This definition was changed to micronutrients provided to children 6–23 months in the last six months after baseline since it was considered a better proxy. The definition provided in the table was the one used in the analysis since it had baseline data.

2/ At baseline the source of verification was the clinical record of children but was changed to vaccination records after baseline since it was considered more comprehensive.

The value of the award was determined on a sliding scale (Table 2), based on the number of points accumulated by the health team every six-month cycle. Teams had to accumulate at least 60 points to receive the minimum incentive of $650 USD worth of goods and were rewarded up to $1,000 USD in goods if they reached 90 or more points per cycle. The experiment was implemented for a total of three six-month cycles.^14^ Teams chose their awards from a list of in-kind incentives created by the MoH which included laptops, air conditioners, microwaves, and other assets that could be used by the team to improve productivity and comfort in their place of work. The awards were assigned to the health unit, not team members, as they were property of the MoH.

**Table 2:** Sliding scale to determine the amount of the incentive obtained in each cycle.

Points obtained^1/^	% of Incentive	Incentive amount (USD)
90–100	100%	$1000
80–89	85%	$850
70–79	75%	$750
60–69	65%	$650
59 or less	0%	$0

^1/^ The points obtained are calculated by dividing the total points obtained in each external verification cycle by the total number of points possible, i.e. excluding those indicators with no data and multiplying it times 100.

### 3.2 Performance measurement

Indicators were measured independently every six months using household surveys and medical record reviews. By design, a pre-specified sample of observations was collected for each team to determine whether there was statistical evidence that the community health team had achieved each target using exact binomial tests.^15^ If a target was reached, the team obtained all points corresponding to that indicator. Otherwise it obtained no points. The use of exact hypothesis tests on a binary outcome of reaching each target was motivated by operational considerations, since establishing sufficiently precise point-estimates to reward teams based on partial achievement of the target would have required prohibitively costly sample sizes for each catchment area.

Table 1 includes the source of verification used for each of the eleven performance indicators. The data source was selected based on feasibility of obtaining a sample in each verification cycle. Household surveys were required to verify most indicators related to utilization and community outreach. On the other hand, medical record reviews were preferred for most quality of care indicators and timeliness of care indicators. Some exceptions were indicators related to narrow population groups such as institutional delivery or children between 12 and 23 months that received MMR vaccination, which due to sample size considerations,^16^ required a proxy measure from medical records.

### 3.3 Performance feedback

**Fig. 1:**
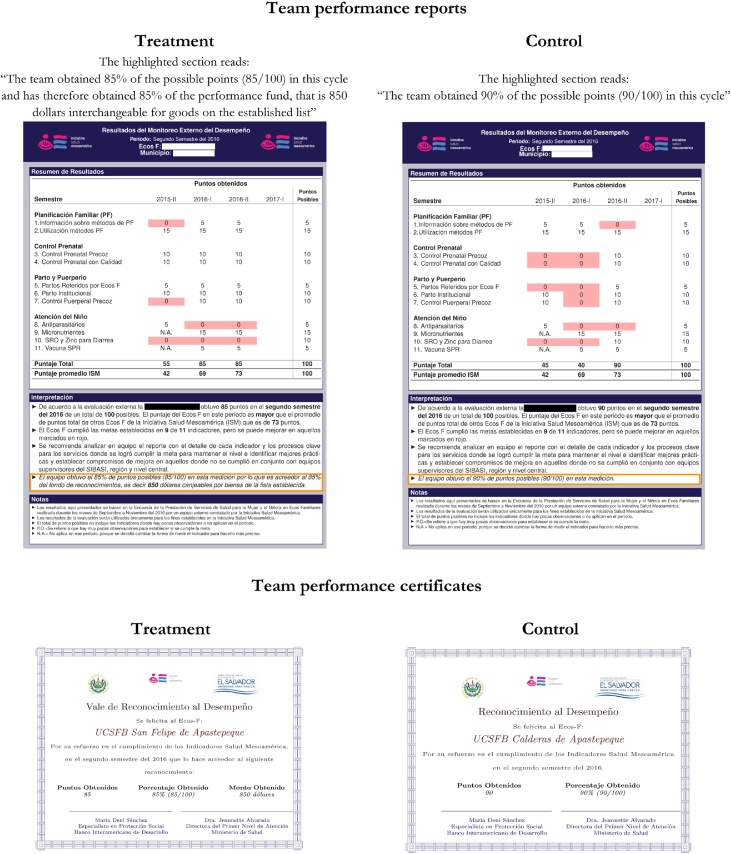
Team performance reports & certificates by treatment status. Notes: The top part of the figure presents a sample of the cover page of the performance report for each team. Highlighted in orange are the differences between the two. The bottom part of the figure presents a sample of the certificates of performance that were provided to teams that obtained 60% of more of the total points possible. The differences between the two are the amount obtained in treatment teams and the title of the certificate (a voucher in the case of treatment and just certificate for control). The identity of the teams was masked for confidentiality purposes.

The results of each independent verification cycle were shared with teams in an individually tailored report. The report included the points obtained for each indicator and the details of the measurement of each indicator, including its definition and data source. Reports were provided for each of the 4 verification cycles to all 75 teams, independent of treatment status. Their design was identical, except that teams assigned to the treatment group had a legend explaining the incentive amount obtained during that cycle. Teams assigned to control had only a description of the points obtained overall, with no mention of incentives (Fig. 1). A full sample report is included in the Appendix.

Reports were delivered at an event held at the end of each verification cycle, with the presence of representatives of each team, as well as MoH authorities. During those events, aggregated results at the department or municipality level were presented and discussed. In addition, teams that achieved 60% of points or higher^17^ received a certificate and public recognition from central MoH authorities. The communication of the results focused on team’s performance without distinguishing between treatment status. There were four verification cycles in total. The first was conducted at baseline, prior to random assignment of teams to treatment and control groups. No certificates were provided at the end of the baseline (only the reports). The other three cycles were performed every six months. A timeline with the dates of each data collection period and performance event can be found in the Appendix.

## 4 Data

Four rounds of household surveys and medical record reviews were collected in the catchment areas of each of the 75 participating community health teams between September 2015 (baseline) and March 2017. Follow-up surveys were collected at the end of each verification cycle, in March 2016, September 2016 and March 2017. Independent samples were obtained for each cycle, producing a panel of repeated cross-sections for each catchment area.

The household survey captured information on sociodemographic characteristics, family planning, and women and children’s health care utilization and behaviors. Surveys were applied to a stratified random sample of 30 dwellings in each catchment area. For each selected dwelling, up to two women ages 15–49 years old were selected at random for the woman and child health modules.^18^ Data on maternal health were collected for each woman’s most recent birth. On average, 2308 dwellings were surveyed for each verification cycle of which 1447 had reproductive age women and 781 had children under five.

Medical record reviews were used to measure targets for population sub-groups that represented a small percentage of the population, namely recent deliveries, pregnancies and children 6–23 months old. The target sample for each catchment area was 17 records of deliveries, 17 of pregnant women and 18 of children 6–23 months old.^19^ Medical record reviews for recent deliveries captured data on prenatal care, delivery and postnatal care. For pregnant women, data were collected on the dates of prenatal care visits and the date of last menstruation, providing a measure of timeliness of prenatal care. Finally, the medical record review for children ages 6–23 months captured MMR (measles, mumps, and rubella) and BCG (tuberculosis) vaccinations, as well as prescription and delivery of micronutrient supplements aimed at reducing the prevalence of anemia. Overall, an independent random sample of 806 records was obtained for women with recent deliveries, 1159 records for pregnant women, and 1336 records for children 6–23 months old.

Household survey and medical records data was used to construct 11 health care performance indicators for each team as described in Table 1. Population-based measures from household surveys were used for indicators on family planning, consumption of deworming pills and knowledge of treatment of diarrhea with ORS and zinc. For all others, medical records were used due to data quality and sample size considerations.^20^ The construction of each indicator is explained in Table A2 in the Appendix. For purposes of our analysis the performance indicators are used (proxies and direct). A discussion and analysis comparing results from proxies to those of direct outcomes is presented in Section 7.

Finally, although the objective was to collect data for all 75 health teams in each verification cycle, the surveyors were unable to collect the household survey or medical records in some catchment areas, primarily due to security concerns with gang violence, which is prevalent in the intervention areas. We have complete data (all four cycles for all 11 indicators) for 64 out of 75 catchment areas. We use this as our main analysis sample. Table A3 in the Appendix presents a breakout of the sample by source and verification cycle. As discussed in Section 7, we found no evidence of a relationship between experimental assignment and attrition.

## 5 Identification and estimation strategy

### 5.1 Identification and experimental design

Our experimental design offers plausibly causal estimates of the effects of the in-kind incentives on provider performance. First, our sample is composed exclusively of existing MoH community health teams. Since teams were constituted by the MoH prior to the start of the incentive pilot, we avoid selection of individual health workers into teams. Moreover, all teams were subject to the same MoH institutional environment, including compensation rules. Second, random assignment of teams to treatment and control groups reduces the potential of selection on unobservables. Finally, all teams received identical measurement, performance feedback, and public recognition for their achievements, allowing us to credibly isolate the effects of the in-kind incentives.

Our sample is composed of 75 community health teams^21^ within 8 administrative regions in 14 municipalities. MoH’s administrative regions are responsible for the network of primary and secondary units in a pre-defined area including supplying inputs, supervision, and management. We implemented a stratified random assignment procedure within three blocks, two rural and one urban. The two rural blocks of about the same size were constructed based on the administrative regions.^22^ All urban teams were grouped into one block. Blocking was done to guarantee balance within each administrative region and to improve precision (List et al., 2010). A summary of the number of health teams by block is presented in Table A4 in the Appendix.

The random assignment was conducted in a public event in October 2015, with representatives of all health teams and national authorities, in which the incentive scheme was presented along with the random assignment procedure. Teams were assigned at random^23^ to one of two phases.^24^ Phase I teams (treatment group) were eligible for the incentive scheme during the 6^th^, 12^th^, and 18^th^-month verification cycles, whereas phase II teams (control group) would only be eligible for the incentive scheme during the 18^th^-month verification cycle (see timeline in the Appendix). Since teams in the control group were aware of their pending incorporation into the incentive scheme as of the 18^th^ month verification cycle, one threat to identification in this phase-in design is that control teams, in anticipation of future incentives, could start working to improve their performance since the start of the experiment. If this mechanism is at work, then our estimates of the incentive effects would be attenuated. We discuss this and other threats to identification in Section 7.

### 5.2 Estimation strategy

To estimate the effect of incentives we estimate the following regression:

(1) $$Y_{ijbt}=\delta T_{jb}+\varphi_{b}+\beta X_{ijbt_{0}}+\varepsilon_{ijbt}$$

where $Y_{ijbt}$ is the outcome of interest for individual i, in the catchment area of team j, of block b, in time t; $T_{jb}$ is an indicator equal to one if team j in block b was assigned to treatment; $\;\varphi_{b}$ are block effects; $X_{ijbt_{0}}$ represents baseline covariates; and $\varepsilon_{ijbt}$ is an error term, which is clustered at the catchment area level. The parameter of interest is δ, which captures the average difference between treatment and control groups within each block. Under standard identification assumptions, that is, if treatment assignment is independent of the outcomes of interest, then, δ, will capture the effect of in-kind incentives. While this assumption is untestable, we provide evidence that randomization was successful in producing balance for a comprehensive set of health team, patient and catchment area characteristics presented in Table A5 in the Appendix. The balance test was performed by estimating Eq. (1), without baseline covariates, on a set of baseline variables. In addition to this balance test we also include similar balance tests for all the outcomes analyzed in the results section.

Our preferred regression estimate of δ pools data from the 6^th^ and 12^th^ month follow-up rounds (during which the control group was not eligible for incentives) and includes controls for team-level outcomes of interest at baseline, $Y_{jbt_{0}}$. We pool follow-up rounds to increase the sample size and reduce variance (McKenzie, 2012) and we include the baseline outcome as a control to improve statistical power (McConnell and Vera-Hérnandez, 2015) and to account for chance differences between groups. As a robustness check we also compare the estimates of our main outcomes with those using differences-in-differences and present disaggregated effects by follow-up round in the Online Appendix.

Our primary outcomes are the 11 indicators used to measure performance which are grouped into four domains: community outreach, quality of care, timeliness of care, and utilization. We present individual estimates for each outcome in the Online Appendix. The grouping of indicators into each of the domains follows Table 1. The indices on each domain are average standardized treatment effects (ASTE), which are defined as follows:

(2) $${ASTE}_{d}=\frac{1}{K}\sum_{k\varepsilon K}\frac{{\hat{\delta }}_{k}}{{\hat{\sigma }}_{k}}$$

where ${ASTE}_{d}$ is the average standardized treatment effect for domain d, which is composed of K outcomes, ${\hat{\delta }}_{k}$ is the treatment effect for outcome k estimated from Eq. (1) and ${\hat{\sigma }}_{k}$ is the standard deviation of the control on outcome k. Using this approach, which is akin to that used by Kling et al. (2004); Spenkuch (2012), and Clingingsmith et al. (2009) has several advantages. First, since it aggregates the effect of different measures within a domain, it increases statistical power. Second, it reduces the number of hypothesis test performed on the main analysis from 11 to 4, and therefore reduces the likelihood that we find effects just by chance (Type I error). Finally, it allows us to combine effects from outcomes from different sources (medical records, household surveys) and population groups into a single metric. We follow Kling and Liebman (2004) in estimating the standard errors for ASTE for each domain and use a seemingly unrelated regression framework to account for covariance across estimates.^25^

## 6 Results

### 6.1 Overall performance score

**Fig. 2:**
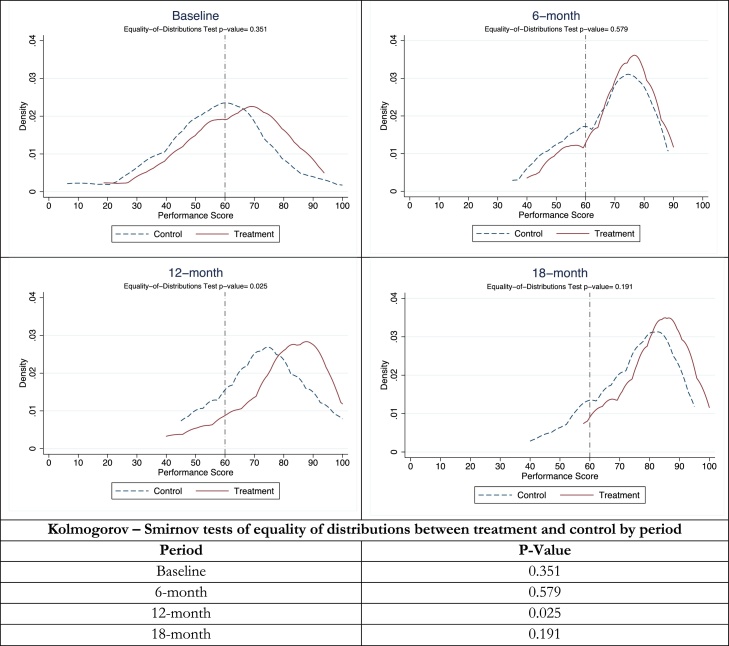
Distribution of team performance scores by treatment status and time period. Notes: The graphs present the kernel density of the distribution of the performance score of all teams by treatment assignment. The score is calculated as explained in Section 3 and was the one informed to teams during the period. The exact p-value of the Kolmogorov-Smirnov test of equality of the treatment and control distributions in each period is presented on the second column. During the 6^th^ and 12^th^ month follow-ups the experimental only treatment teams received incentives. At the 18^th^ month follow-up both treatment and control teams were eligible for incentives.

Fig. 2 presents the kernel density plots of health team’s performance scores by time period (baseline, 6-month, 12-month and 18-month) and treatment status. The overall performance score is the aggregate weighted measure of targets met by a health team, with weights equal to the points established for each indicator (Table 1). The score determined whether teams in the treatment group received incentives, according to the sliding scale presented in Table 2. It is not a measure of the magnitude of the gains, but rather of compliance with the targets of all indicators and a summary measure that teams received on their performance every cycle. For each period, we perform a Kolmogorov-Smirnov (KS) test of equality of distributions between treatment and control and include the p-values of these tests at the bottom of Fig. 2.

Fig. 2 reveals several findings. First, there are no statistical differences between the distributions at baseline and six-months, the first period where treatment teams were eligible for incentives. Differences appear 12-months after the scheme was introduced, as seen both in the figure and confirmed with the result of the KS test. Second, treatment and control distributions both shifted right over time, indicating improvement after the scheme was in place. Third, while at baseline about half of all teams were below the threshold of eligibility for incentives (60 points), only a small share remained below this threshold 12-months after baseline. This is evidence that low performance teams at baseline had a marked improvement and suggests a sort of “threshold effect” as teams strived to be above the 60 percent threshold from which incentives and public recognition were given. While Fig. 2 gives a clear picture of team performance, it does not provide an idea of the magnitude of the gains in performance, since it is just a measure of compliance with targets for the 11 indicators. We look in detail for the magnitudes of improvements by domain in the next subsection.

The fact that control teams had a marked improvement in performance during the first six months that mirrored those in the treatment group could have several explanations. First, all teams received a baseline report with their performance, and there is empirical evidence that information on quality of care provided to physicians can improve their performance (Kolstad, 2013). Second, all teams regardless of their experimental assignment received public recognition, which can be an effective motivator in labor relations (Kosfeld and Neckermann, 2011) if they met 60 percent of more the established targets. Moreover, qualitative evidence suggests that public recognition with the certificates was something that motivated community health teams and those not getting one felt compelled to strive harder (Munar et al., 2018).^26^ Third, all teams were part of SMI, which had incentives at the national level. Feedback and support were provided to all teams by municipal and administrative region supervisors to create local plans of improvement aimed at achieving SMI targets. Fourth, an anticipation effect could be at work, since control teams knew they would be eligible for incentives at 18-months. We discuss this further in Section 7.

### 6.2 Main outcomes

The results on the ASTE are presented by domain in Table 3. The first column presents the baseline estimate, the second column the effect without controls and the third the effect controlling for the baseline level of the outcomes of interest. Overall the results from our preferred estimation model (column 3), indicate that the in-kind incentives had positive effects on all contracted domains, with the largest effect on community outreach and quality of care. Below is a brief description of what is measured in each domain, as well as the main result. Estimates of individual indicators can be found in Table A8 in the Appendix.

**Table 3:** Treatment Effect by Domain.

	Baseline	Post-treatment (No controls)	Post-treatment (Controlling for Baseline)
	(1)	(2)	(3)
**ASTE on Contracted Indicators by domain**			
*ASTE Community Outreach*	0.0358	0.1763**	0.1748**
	(0.069)	(0.071)	(0.069)
p-value	[0.603]	[0.013]	[0.011]

*ASTE Quality of Care*	−0.1361	0.1349**	0.1427***
	(0.095)	(0.058)	(0.054)
p-value	[0.152]	[0.020]	[0.008]

*ASTE Timeliness of care*	0.0954	0.1203**	0.1021*
	(0.089)	(0.057)	(0.052)
p-value	[0.286]	[0.035]	[0.051]

*ASTE Utilization*	−0.0323	0.0897**	0.0958**
	(0.065)	(0.041)	(0.040)
p-value	[0.620]	[0.028]	[0.015]

***ASTE Non-Contracted Outcomes***	0.0937*	0.0379	0.0215
	(0.053)	(0.035)	(0.031)
p-value	[0.079]	[0.274]	[0.485]

Community Health Teams	64	64	64

*Notes*: The analysis sample is the 64 teams with data in all waves. For each domain, the table presents the estimate of the average standardized treatment effect (ASTE) as described in Eq. (2) using the individual outcomes as described in Table 1. Column (1) presents the estimate of δ from baseline on the outcome of interest using Eq. (1),but excluding any baseline covariates. Column (2) presents the estimate of δ using the pooled 6^th^ and 12^th^ month follow-up data excluding baseline covariates. Column (3) presents the same estimate as (3) but includes as baseline covariate the mean team level outcome of interest at baseline. All estimates are with individual level data. Details of the sample of interest for each indicator is in Table A8 in the Appendix. Standard errors are clustered at the team level and are presented in parenthesis. P-values are included in square brackets.

*p < 0.10, ** p < 0.05, *** p < 0.01.

#### 6.2.1 Community outreach

The ASTE on community outreach includes the effect on two outcomes measured from the household survey. The first, information on modern family planning, refers to women 15–49 years old that declare to have received information on modern family planning methods^27^ by health personnel during the last six months. The second is knowledge by mothers of children under five on treatment of diarrhea with oral rehydration salts (ORS) and zinc. Treatment of children’s diarrhea with zinc in addition to ORS is recommended by the WHO, since it reduces the severity and duration of diarrhea episodes among children in low-resource settings (Lazzerini and Wanzira, 2016). The outcome is relevant in El Salvador since only about half of baseline diarrhea cases sought medical treatment and zinc had been introduced recently, in 2014, as part of the country’s clinical guidelines. Overall, in-kind incentives increased community outreach by 0.17 standard deviations. Prior to the introduction of the incentive scheme there was adequate balance (column 1).

#### 6.2.2 Quality of care

The quality of care domain includes two outcomes related to the care received by women during pregnancy at the health facility: quality of prenatal care and reference to institutional delivery. Both are measured from medical records. On average, about 95 percent of pregnant women in the catchment area received prenatal care from the community health teams at MoH facilities. Quality of prenatal care is an indicator equal to one if the pregnant women received all the clinical examinations required by national guidelines during all prenatal care visits.^28^ Required examinations include weight, blood pressure, fundal height (after 14 weeks of gestation), fetal heart rate and fetal movements (the last two required after 20 weeks of gestation). In addition, lab tests of glucose, HIV, hemoglobin and urine should be done at least once during pregnancy. These examinations and tests permit the detection of critical risks during pregnancy including eclampsia, anemia, diabetes, intrauterine growth restriction, and/or HIV, that could lead to increased maternal and fetal morbidity and mortality if undetected and untreated (WHO, 2016; Bhutta et al., 2014). The second outcome of interest is reference to institutional delivery which measures whether the pregnant women received advice on institutional delivery and registered a delivery plan (a plan developed by provider and patient on the place of delivery, transportation and arrangements during pregnancy).

The incentives had a statistically significant effect of 0.14 standard deviations overall in quality of care. It is worth noting that while there is a relatively large, but statistically insignificant ASTE on quality of care at baseline (column 1), it goes in the opposite direction of the treatment effect (column 3) and we have no evidence to believe that this reflects a systematic difference between treatment and control groups.^29^

#### 6.2.3 Timeliness of care

Timeliness of care includes two outcomes: timely antenatal care and timely postnatal care. Both are measured from medical records. The first is an indicator equal to one if the first prenatal care visit occurred during the first trimester of pregnancy. Early prenatal care is part of WHO standards of care (WHO, 2007) since it allows to detect and treat conditions that could compromise fetal and maternal health (Carroli et al., 2001). The second is an indicator equal to one if the first postnatal care visit occurred within a week after delivery. Early postnatal care is a critical part of the continuum of care for pregnant women since it allows detection of complications arising after delivery (WHO, 2013), and more than a third of maternal deaths in the region occur during this period (Kassebaum et al., 2014).

The average treatment effect on timeliness of care is of 0.10 standard deviations. While relatively smaller in magnitude, this domain is harder to influence by teams, as it involves not only clinical knowledge, but also developing strategies to capture women early in pregnancy or right after delivery.

#### 6.2.4 Utilization

The utilization domain includes a total of five indicators. Two of these are measured with household survey data: current use of modern family planning methods by women 15–49 in need of contraception and consumption of two doses of deworming pills by children 12–50 months in the last year. For the other three measures, sampling constraints required constructing proxies from medical records (we compare these proxies to their equivalent household measures in Section 7 for a subset of teams were data are available). These utilization proxies include an indicator for institutional delivery from medical records, an indicator for prescription of micronutrients sachets for children 12–23 months and an indicator for MMR vaccination according to the medical records of children of the same age.^30^

Overall the team incentives were effective in producing a small improvement in the utilization of health services, of 0.096 standard deviations as presented in Table 3. This effect came about by relatively small gains in all five indicators of between three to five percentage points. While most of these individual effects are statistically insignificant, we gain precision by pooling them together in a single measure (see Table A8 in the Appendix).

### 6.3 Non-contracted outcomes

A common concern in the design of pay-for-performance schemes is that if individuals have multiple tasks to complete as part of their job, but only a subset are subject to incentives, in response they could shift effort away from non-contracted outcomes. This unintended effect is commonly referred to as multi-tasking in the theoretical literature of contracts (Holmstrom and Milgrom, 1991). While most of the literature focuses on multitasking at the individual level,^31^ teams could also be subject to this unintended effect if they share a common objective function and are able to coordinate.

We test whether the incentive scheme had this type of unintended consequences by assessing three non-contracted outcomes: detection of diabetes, hypertension, and cervical cancer among women 15–49 years old during the last six months. None of these outcomes were included as part of the incentive scheme, but they were activities to be performed by community health teams in their catchment area. All were constructed from the household survey. We present the average standardized treatment effect at the bottom of Table 3, finding no evidence of shifting of effort from these activities.

### 6.4 Heterogeneity by baseline performance

As discussed in Section 3, the incentive scheme was structured as a step function based on a non-linear sliding scale starting at a threshold of 60 points out of 100 (Table 2). A common concern with this type of design is that if performance is costly, the marginal cost of improving performance could outweigh the benefits for teams far below the threshold. In this case, the incentives might only affect those teams that are slightly below the threshold where the benefits outweigh the costs (Miller and Babiarz, 2014). This type of response would also be expected from the behavioral goal-gradient hypothesis (Kivetz et al., 2006), that states that effort is increased as the distance to the rewards shortens and decreases once the threshold is passed and the reward obtained.

To our knowledge there is limited empirical evidence on this type of response in the pay-for-performance in health literature.^32^ We analyze the response of teams to the incentive scheme by grouping teams into three categories according to their baseline performance. The first group includes 30 teams below the incentive threshold (performance score of 0–59 inclusive). The second group is comprised of 19 teams with baseline performance in the first bracket of incentives (performance score of 60–69). And the third group is comprised of the 15 highest performing teams that scored above the first bracket at baseline (performance score of 70–100).^33^ For each of these groups we analyze the magnitude of improvement on contracted outcomes by estimating the ASTE. This is a more relevant measure than the gains in the performance score, since it reflects the magnitude of gains made by teams in each category and not just the overall compliance with the targets.

**Fig. 3:**
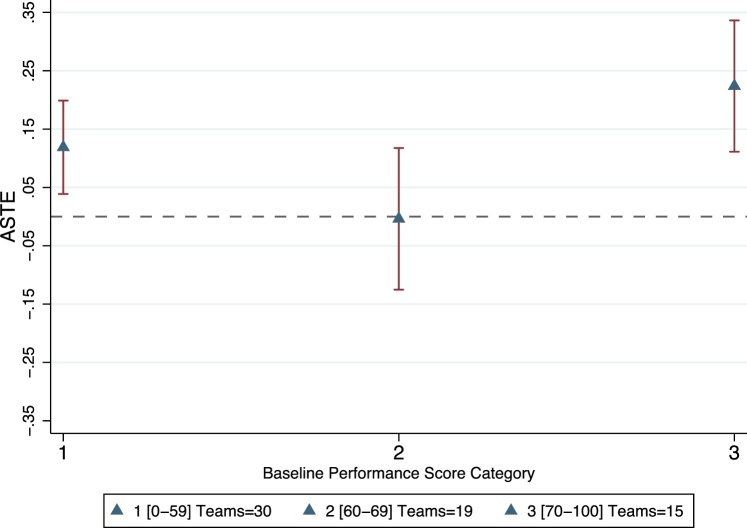
Average standardized treatment effect of all contracted outcomes by category of baseline performance score. Notes: Categories of baseline performance score are constructed based on the sliding scale for incentives. The first category includes teams with a baseline performance below the threshold for incentives, i.e., 60. The second category includes teams just at or above the incentive threshold, i.e. 60–69 inclusive. The third category includes those teams with a baseline performance score of 70 or higher. The number of teams in each category is displayed in the figure label. Average standardized treatment effect of all eleven contracted outcomes, obtained as described in Section 5, using the model that pools both the 6^th^ and 12^th^ month follow-ups and with block effects and the baseline value of the outcome of interest in each regression. Confidence interval at 95% constructed from standard errors clustered at the team level, displayed on the red lines around each estimate.

First, we estimate effects on the pooled ASTE for all 11 contracted outcomes as a single summary measure, using our preferred model that pools the 6^th^ and 12^th^ month measurements and controls for baseline level of each outcome. The results, presented in Fig. 3, suggest that the lowest performance teams experienced substantial gains of about 0.12 standard deviations on the pooled ASTE. In contrast, those teams in the first bracket of 60–69 points at baseline achieved essentially no gains. Finally, the highest performing teams at baseline had the largest effect, achieving on average an increase of 0.22 standard deviations on the 11 contracted outcomes.

Next, we analyze results by domain to understand how teams in each baseline performance group achieved their improvements. This is particularly illustrative as domains could require different levels of effort. The results, presented in Table 4, suggest that teams with the lowest-performance score at baseline, made substantial progress in community outreach and quality of care, with effects of 0.26 and 0.13 standard deviations respectively (Table 4, second column). Teams at the threshold of incentives at baseline show no indication of improvement in any domain. Teams on the high end of the performance distribution at baseline, had large improvements on most domains. Interestingly, these teams were the only ones that achieved improvements in the timeliness of care and utilization domains (with 0.29 and 0.19 standard deviations respectively).

**Table 4:** Heterogeneity of treatment effects by performance score at baseline.

		Post-treatment (Controlling for Baseline)		
		Baseline Performance Score Category		
	Overall	0-59	60–69	70-100
	(1)	(2)	(3)	(4)
**ASTE on Contracted Indicators by domain**				
*ASTE Community Outreach*	0.1748**	0.2615***	−0.1132	0.3374**
	(0.069)	(0.096)	(0.105)	(0.141)
p-value	[0.011]	[0.006]	[0.280]	[0.017]

*ASTE Quality of Care*	0.1427***	0.1330*	0.0706	0.1268
	(0.054)	(0.071)	(0.090)	(0.119)
p-value	[0.008]	[0.061]	[0.431]	[0.288]

*ASTE Timeliness of care*	0.1021*	0.0859	−0.0328	0.2854***
	(0.052)	(0.058)	(0.139)	(0.093)
p-value	[0.051]	[0.140]	[0.814]	[0.002]

*ASTE Utilization*	0.0958**	0.0691	0.0217	0.1925***
	(0.040)	(0.070)	(0.067)	(0.049)
p-value	[0.015]	[0.326]	[0.747]	[0.000]

**ASTE Non-Contracted Outcomes**	0.0215	0.0419	−0.0074	0.0070
	(0.031)	(0.049)	(0.073)	(0.037)
p-value	[0.485]	[0.387]	[0.919]	[0.852]

Community Health Teams	64	30	19	15

*Notes*: The sample of analysis is of the 64 teams with data on all waves. Column (1) presents the estimates of the average standardized treatment effect (ASTE) post-treatment controlling for team-level baseline outcomes by domain, as presented on Panel A, Column (4) of Tables 3 through 7. Column (2) presents results for teams below the cutoff for incentives in their performance score at baseline. Column (3) presents results for teams with a baseline performance score of 60–69, which is equivalent for the first bracket of the incentive scheme. Column (4) presents results for teams with the highest levels of performance score at baseline (70–100). The performance score is defined as in Section 3. Standard errors are clustered at the team level and are presented in parenthesis. P-values are included in square brackets.

*p < 0.10, ** p < 0.05, *** p < 0.01.

The performance gains amongst low-baseline-performance teams were concentrated on domains that required less team coordination. For example, community outreach was mainly the responsibility of community health workers, whereas quality of care relied on physicians and professional nurses. On the other hand, high-baseline-performance teams also achieved gains on domains that required more team coordination, such as utilization and timeliness of care. These domains require that health teams coordinate closely to change patient behaviors and follow-up with patients. These results may reflect that high- baseline-performance teams were more closely articulated to begin with and were thus able to respond to incentives on the more complex domains. Finally, as in the aggregate analysis, we find no evidence of heterogeneity of shifting efforts away from non-contracted outcomes for any sub-groups, with small and statistically insignificant coefficients on non-contracted outcomes.

Overall, these results suggest that the in-kind incentives were sufficiently attractive to motivate higher performance for low-baseline-performance teams, however these improvements were concentrated in domains that required relatively less team effort. If all teams were purely maximizing their return to effort, and effort is costly, it might be expected that those above the incentive’s threshold would do minimal or no additional effort. Our results are consistent with this hypothesis for teams at the threshold of 60–69 points at baseline which show no indication of responding to the incentive scheme. However, the large effects we find on high-performing teams seems at odds with the theoretical prediction. High-performers appear to increase their effort substantially across all domains, including the costliest in terms of team coordination. One potential explanation is that that they were the most productive teams to begin with, so small changes in effort could be translated into large changes in performance. An alternative explanation is that for these teams, reaching the highest incentive amount could have served as a goal, particularly since they had the lowest distance to the highest possible amount. If this was the case, a behavioral response consistent with the goal-gradient hypothesis would be that they increased their effort as they saw this goal within reach, even if it was costly, as they were highly motivated to reach the maximum incentive possible. Finally, the U-shaped observed on the response to the incentive-scheme is similar to that observed in response to rank-order feedback in lab settings and has been described as “first-place loving” and “last-place loathing” (Gill et al., 2018). While rank-order feedback was provided to all teams,^34^ the in-kind incentive effect could have been additive to that of rank-order feedback and hence the results we observe.

## 7 Competing explanations

### 7.1 Cheating

Incentive schemes are fraught with potential unintended effects, particularly for complex tasks for which there are only imperfect measures of effort. A common concern is that individuals might focus on low-effort activities to obtain the incentive without influencing the underlying outcome of interest.^35^ The performance scheme we study here uses two different sources to measure performance: a household survey and a medical record review. We have no reason to believe that the household survey could be easily manipulated, and medical records are legal documents that if tampered can lead to legal action. However, we may still be concerned that teams tampered with medical records to obtain short-terms gains, particularly since medical records were used to measure performance on seven out of the eleven performance indicators. If this were the case, then part of our effects could be due to changes in reporting rather than improvement in the underlying population outcomes.

To assess this possibility, we compare treatment effects for a subset of outcomes in which we have comparable indicators from medical records and household surveys. Due to the design of the sampling scheme, we have data from both sources for four outcomes in 33 catchment areas, with comparable cohorts of children and pregnant women.^36^ These outcomes are timely prenatal and postnatal care, institutional delivery, and MMR vaccination. The samples of medical records and household surveys were independent, so they do not reflect concordance of data by patient, but rather an estimate of the outcome of interest among the population of the catchment area of the team.^37^

In Table 5 we present results using outcomes from each source, and test whether there are statistically significant differences between them. The results of these tests (column 5) indicate that there are no statistically significant differences between data sources, and more importantly, the size of the effects is quite similar for most outcomes. The similar magnitudes are relevant, since we have relatively low power for these tests given the reduced sample of catchment areas. The only outcome in which the effect from the medical records proxy is larger than that of the household surveys is postnatal care (0.12 Vs. 0.056 percentage points). However, the baseline level of this indicator was lower in medical records compared to the household survey (61% Vs. 92%). For a substantial number of medical records (14 percent at baseline) there was no evidence of the post-partum care in the record since it only included actions taken by physicians and nurses and not those by community health workers. Clinical guidelines allowed community health workers to perform the early postnatal checkup, since women usually remained at home after delivery. Since the population-based coverage of early postnatal care was high at baseline (92 percent), and we find a significant effect of around 6 percentage points in this measure, the higher effect on the medical record is most likely a catch-up of reporting of services provided to the population. Similar issues occurred for other outcomes in which the medical record measures were substantially lower than the population-based measures (columns 1 and 3 of Table 5).^38^

**Table 5:** Comparison of individual outcome effects by source.

	Common Sample of Units & Time-Periods				
	Medical Records Proxy		Household Survey		
	Control Mean	Post-treatment (Controlling for Baseline)	Control Mean	Post-treatment (Controlling for Baseline)	P-value of difference between (2) and (4)
	(1)	(2)	(3)	(4)	(5)
**Timeliness of Care**					
*Timely Prenatal Care*	0.7109	0.0259	0.5746	−0.0000	
		(0.034)		(0.072)	
p-value		[0.450]		[1.000]	[0.717]
N		927		276	
Units		33		33	

*Timely Post-natal Care*	0.6080	0.1213**	0.9167	0.0563**	
		(0.051)		(0.021)	
p-value		[0.025]		[0.012]	[0.228]
N		675		275	
Units		33		33	

**Coverage**					
*Institutional Delivery*	0.7557	0.0307	0.9167	0.0068	
		(0.037)		(0.031)	
p-value		[0.414]		[0.829]	[0.566]
N		675		276	
Units		33		33	

*MMR Vaccination*	0.6364	0.0538	0.8611	0.0752	
		(0.051)		(0.062)	
p-value		[0.295]		[0.235]	[0.803]
N		1,105		154	
Units		33		33	

*Notes*: The analysis sample is 33 teams with data in all waves for both households and medical records. All outcomes include common cohorts of patients for medical records and household surveys. Columns (2) and (4) present the estimates of the treatment effect (ASTE) post-treatment controlling for the team-level baseline of the outcome. Standard errors are clustered at the team level and are presented in parenthesis. P-values are included in square brackets.

*p < 0.10, ** p < 0.05, *** p < 0.01.

Taken together, the evidence suggests that there were not systematic differences between data sources, supporting the claim that teams did not tamper with records. Two aspects of the incentive scheme may have helped limit tampering. First, teams were aware that performance would be measured using both medical records and independent household surveys. The latter may have been perceived as an audit on work reported in medical records. Second, the MoH conducted close supervision from the administrative regions and the central level for all teams, meeting every three months to discuss performance, understand barriers to achieve targets, and determine improvement plans. While this is standard practice at the national level, it may have been particularly salient for the 75 catchment areas studied here given their relevance to the national results-based aid model under SMI.

### 7.2 Anticipation and frustration effects

Due to the lack of blinding in the experiment, health teams in the control group were aware of their eligibility for incentives after the initial 12-month experimental period. In this context, a threat to identification could be anticipation or frustration effects which result in control teams increasing or decreasing their performance prior to becoming eligible for incentives. As observed in Fig. 2, there is a clear improvement in control team performance relative to baseline during the first 12 months. This trend rules out frustration effects, but it could be explained by the effects of information and recognition for which controls were eligible. Unfortunately, we are unable to separate anticipation effects from information and recognition effects. If anticipation did play a role in boosting performance for the control group, our treatment estimates would be attenuated downward. Since we do find positive and statistically significant effects for all domains analyzed, we interpret our estimates as lower-bounds of the effect of in-kind incentives in the presence of anticipation effects.

### 7.3 Additional robustness checks

Section 2 in the Appendix presents complementary robustness checks that help us rule out competing explanations for the observed results. First, we check for evidence of differential attrition. We then control for time invariant unobserved team level characteristics through difference in difference models. Finally, we rule out effects of the in-kind incentives on productivity. In all cases, the complementary analysis supports the validity and our interpretation of the main findings.

## 8 Discussion

Improving the quality of health care is a global challenge that is particularly salient in the poorest and hardest to reach areas in low and middle-income countries. Pay for performance is a promising tool to align health provider’s incentives more closely with health outcomes. Yet despite growing interest, causal evidence is mixed, and only a few studies isolate the effect of incentives separate from information, feedback and/or recognition. Moreover, the design of incentive schemes, such as whether to pay cash or in-kind incentives and individual versus group incentives likely plays a key role in their effectiveness, unintended consequences, and sustainability, yet we know little about the plusses and minuses of different models. In the health sector providers may be at least partially motivated by intrinsic factors, and payment of monetary incentives could face institutional barriers that make them untenable in the public sector. As such, how to structure a pay for performance incentive scheme is context-specific and remains largely an open question (Miller and Babiarz, 2014).

We present experimental evidence on the effect of performance-based in-kind incentives for community health teams. Conventional economic theory suggests that in-kind and group incentives may be relatively low-powered compared to cash or individual incentives, since benefits are shared by team members and may not align with individual worker preferences, and there is a risk of free-riding among team members. Yet our results suggest in-kind incentives for teams led to substantial improvements in performance across multiple domains. The incentives even led to changes in patients’ behaviors, which are not under the full control of providers, such as timeliness of care and utilization of health services. While all teams (treatment and control) improved their performance over time, those assigned randomly to receive incentives first had a faster rate of improvement, with these gains concentrated amongst the lowest and highest performers at baseline.

It appears unlikely that increased performance on incentivized outcomes was the result of shifting away effort from non-contracted outcomes^39^ or from gaming the system. The latter despite ample room to simply improve reporting since some proxy measures in medical records tended to be under-reported as compared to those from the household survey. Qualitative evidence suggests that a key element to avoid these unintended consequences was that monitoring and supervision by the MoH focused on the full portfolio of services provided by teams and feedback on the performance reports centered on how to change the underlying outcomes rather than the proxies used for measuring performance. In addition, the fact that every verification cycle collected data from both household surveys and medical records might have reduced the temptation of tampering medical records.

Qualitative evidence suggests that while the performance reports and certificates created a sense of competition between teams regardless of their experimental assignment, those eligible for the in-kind incentives were able to motivate all team members to go the extra mile and perform additional home visits, work on weekends, and put in additional effort to achieve their targets and develop creative ways to influence patients’ behaviors. For instance, some treatment teams came up with ideas such as informing mothers of the market price of micronutrients sachets (which are provided for free by teams) to improve mothers’ valuation of them. Moreover, it seems that in-kind incentives were valued by teams more like an award and recognition (particularly since they were announced in front of their peers) rather than for their material value, which might elicit greater effort (Kosfeld and Neckerman, 2011). As a team member put it when discussing the in-kind incentives: “*That's like a bonus for what we were already doing. So, let's try harder. Since they are recognizing our work, and they want to recognize it, well, then it was like a motivation, an extra incentive to strive more, right? To work*” (Munar et al., 2018). Overall, our results suggest that in-kind group incentives could be a powerful tool to improve health worker performance and a viable alternative to monetary incentives in certain contexts.

## Funding

This work was supported by the Bill and Melinda Gates Foundation; the Carlos Slim Foundation; and the Spanish Agency for International Development Cooperation. Funders had no role in study design, data collection and analysis, decision to publish, or preparation of the manuscript.

## Footnotes

1 Pay-for-performance experiments with credible attribution include Basinga et al., 2011; Bonfrer et al., 2013; de Walque et al., 2015; Shen et al., 2017; Celhay et al., 2018. The Health Results Innovation Trust Fund is undertaking impact evaluations of performance-based financing in health interventions in over 20 countries, primarily concentrated in Sub-Saharan Africa (http://www.rbfhealth.org/).

2 Our findings also relate to the literature of awards, which are types bundled with some form of material benefit or prize (Kosfeld and Neckermann, 2011) and to the literature on social recognition for public workers (Gauri et al., 2018). In our setting we are able to identify the added-value of providing in-kind incentives for teams in addition to social recognition.

3 This is more so if incentives are for teams, as different team members might have different preferences for specific in-kind incentives while monetary incentives are fungible and could be easily divided among team members.

4 The backfiring of monetary incentives might be more relevant in pro-social tasks liking blood donations (Lacetera and Macis, 2010). However, in some type of government work, including public health, tasks might be perceived similarly as pro-social if they have wider social benefits beyond those enjoyed by employers and employees (Ashraf et al., 2014).

5 Ashraf, Bandiera and Jack (2014) estimate the effect of pure non-monetary incentives (a star stamp for each sale) and compare it to that of financial rewards based on sales, but they do not test the effect of an in-kind incentive.

6 This section relies on MINSAL (2010), which provides a comprehensive account of the Health Reform in El Salvador and on MINSAL (2011), which details the guidelines of care of community health teams.

7 The term for these teams in Spanish is *Equipos Comunitarios de Salud Familiar*, or *ECOS-F.*

8 The Ministry of Health (MoH) was the main provider of health services in these areas. For example, according to UNICEF’s Multiple Indicator Cluster Survey (2014), in 2014, the MoH provided around 93 percent of postnatal care services in rural areas and close to 75 percent in urban areas.

9 Well-visits are preventive visits scheduled periodically. For children they are more frequent when they are younger and become less frequent as they age. A usual child well-visit includes a complete physical exam, weight and height measurements to track growth and some screening tests. Nutritional advice is also usually provided during these visits.

10 The public-private partnership combined funding from the Bill & Melinda Gates Foundation, the Carlos Slim Foundation and the Government of Spain that was managed by the Inter-American Development Bank.

11 For the purposes of SMI the Mesoamerican region included Belize, Guatemala, El Salvador, Honduras, Nicaragua, Costa Rica, Panama, and the state of Chiapas in Mexico.

12 In 2011, prior to the start of SMI, around 85.4 percent of care sought by women was provided by public facilities and by 2016 (IHME, 2011), the figure was close to 92 percent, of which around 97% was provided at MoH facilities.

13 While the design originally was set for two phases, a third phase was approved in 2018. Targets were independently verified through a combination of household and facility surveys (Mokdad et al., 2015).

14 The total incentive amount was initially set at $1000 dollars for each of 4 verification cycles excluding baseline. However, due to time constraints in the financing of the pilot, only 3 verification cycles (plus baseline) were possible. Incentives for the final two cycles were combined, and teams were granted up to $2000 in incentives in the third and final cycle.

15 The tests were performed to assess the null hypothesis that the team had achieved the target established for the indicator. Due to the small sample sizes for each team, the test was performed based on the binomial distribution rather than a continuous distribution approximation. These tests were useful to identify those teams that were performing well below the target.

16 The sampling strategy is discussed in more detail in Section 4.

17 The percentage was obtained by adding the total points obtained in a verification cycle and dividing them by the total points from indicators that could be measured in that cycle. This was done, since in some cycles, for reasons beyond the control of the teams, there was not enough sample to estimate compliance with the threshold, and hence the points from this indicator were not counted towards the total. One of the most common reasons for no data availability was the security situation in certain areas, where the data collection team could not enter.

18 The sample of 30 dwellings per catchment area was designed so that, in expectation, two thirds of dwellings included women between the ages of 15 to 49, and about one-third of dwellings had children under five. This was done to achieve the minimum necessary sample sizes required to distinguish whether a target had been met or not for the population sub-groups of interest, such as use of family planning methods for reproductive age women or consumption of deworming medication for children. All cycles of data selected at most two women ages 15 to 49 except for the baseline, where only one woman was selected at random.

19 If fewer records were located in the reference period, then all records were selected. Since deliveries are a rare event, the actual average sample per catchment area was well below the target.

20 For the indicators of prenatal care quality and timeliness, medical records were preferred since they were more precise in terms of exact dates and procedures performed. Even though household surveys that rely on maternal recall can be accurate for some specific interventions during delivery (Chen et al., 2014) and for conditions during pregnancy (Krakowiak et al., 2015), to our knowledge its reliability in terms of specific clinical items during prenatal care is not that well studied. Moreover, differential recall bias related to pregnancy outcomes and women characteristics has long been known to be a concern in the epidemiological literature (Yawn et al., 1998).

21 All community health teams participated independently in the randomization, except for the two urban teams of Sensuntepeque, which were considered as one for the purpose of random assignment since the two teams shared the same premises. This was not the case for all other teams.

22 These regions are known as *Sistemas Básicos de Salud Integral* (SIBASI) in the country.

23 Random assignment was done by introducing into an opaque box an equal number of marked balls with the numbers I and II (and totaling the amount of teams within a block), shuffling the box and then drawing a ball at random for each team within a block. Teams that selected a ball marked with I were assigned to Stage I (treatment) whereas those with a ball marked with II were assigned to Stage II (control). This procedure was repeated for the three blocks.

24 Teams were informed that due to budgetary constraints the incentive scheme would be introduced in phases, but to give all teams the same chance to be included in the first phase a random assignment would be performed.

25 We also estimated bootstrapped standard errors clustered by community health team and stratified by block with 1000 replications to account for an unknown form of correlation within teams and across outcomes. The results are essentially the same. Moreover, the magnitude of our estimate of standard errors using the Kling and Liebman (2004) approach was slightly more conservative than those of the bootstrap for our main results.

26 As stated by one of the participants in the feedback events regarding the performance certificates *“When we go to the meetings, and I love that…. when we are all there, they say, ‘We are going to give this diploma to the teams that made a great work.’ They call them up, and it looks like they are graduating from something. And my colleagues become really motivated. And maybe you can hear from those who didn’t receive anything phrases like ‘I will propose something for the next meeting because I want to be there.’”* (Munar et al., 2018).

27 This refers to women in need of contraception which are defined as those age 15 to 49, excluding those sterilized, in menopause, that declare to be virgin or not sexually active, and those pregnant or trying to conceive. Modern family planning methods include injectable, contraceptive pills, female or male sterilization, intra uterine devices (IUD), implants, emergency contraception, female or male condoms, contraceptive diaphragm and sponges.

28 On average women received around 4 prenatal care visits in MoH facilities in the 14 participating municipalities.

29 Balance tests are presented in Table A5 in the Appendix, and those presented for all of the other domains are included in the first column of Table 3. Balance tests for each individual outcome are included in Table A8 in the Appendix. Moreover, our preferred model on Column (3) controls for baseline levels, which should account for any chance imbalance, and is more conservative given the direction of the imbalance compared to the difference-in-difference estimate (see Table A10 in the Appendix).

30 For micronutrients and MMR vaccination this was the source of verification for performance during baseline, but was later changed to children with the prescription of micronutrients filled by the team for micronutrients and MMR vaccination according to vaccination booklets, since they were more up to date. We use the original measures since those were those collected in all four verification cycles.

31 The empirical evidence on the existence of this effect in pay-for-performance schemes in health is mixed, most likely as it is contract-specific and also since it would depend on the degree in which contracted outcomes share inputs with non-contracted ones (Miller and Babiarz, 2014)

32 There is some evidence of this individual behavior in marketing from Kivetz et al. (2006) that study the context of consumer reward programs. There is also some evidence on this effect from the literature on non-monetary incentives for prosocial behavior in the study of blood donations (Lacetera and Macis, 2010).

33 As a robustness check we conducted the heterogeneity analysis by quintiles of baseline performance score, finding essentially the same results discussed in this section, albeit treatment effects had wider confidence intervals with fewer observations per group.

34 Rank-order feedback was provided in public at the time performance certificates were awarded. Those not earning a certificate, that is below the 60 points threshold, did not receive their specific rank, but by not receiving the certificate they were informed effectively that they were the lowest performers.

35 For instance, incentivizing teachers’ performance based on their students’ test scores could have unintended consequences such as inflating test scores (Koretz, 2002), exempting the worst students from taking the test (Cullen and Reback, 2006) and even changing students’ answers (Jacob and Levitt, 2003), rather than improving students’ learning and achievement. In the health context, vaccinations could be over-reported if incentives are conditioned on the number of children vaccinated, without actually improving the coverage of immunity among the population (Lim et al., 2008).

36 For this subset of teams, we have adequate balance and we have no evidence of differential attrition by treatment assignment as presented in Tables A6 & A7 in the Appendix, which are similar tests to those presented in Section 7.

37 The outcomes presented in Table 5 are related to maternal care and vaccination, all of which the main provider in the catchment area were the community health teams. For instance, prenatal care was sought in MoH facilities by 95 percent of women with a pregnancy on average over all 75 community health teams.

38 For institutional delivery, the issue was that there was a gap in registration that occurred since deliveries were not performed by teams, but by hospitals which had to send the information on deliveries back to teams and this caused a delay in registration. For vaccinations, it was a similar issue to that of postnatal care.

39 At least from those we measure from women in fertile age related to the prevention of chronic conditions.
