# Supplementary material for: In-kind incentives and health worker performance: Experimental evidence from El Salvador
Source: J Health Econ. 2020 Mar;70:102267. doi: 10.1016/j.jhealeco.2019.102267 (PMC7188218; doi:10.1016/j.jhealeco.2019.102267)
Supplement: Supplementary file 1 [file mmc1.pdf]

Appendix for: In-Kind Incentives and Health Worker Performance: Experimental Evidence from El Salvador

By Pedro Bernal and Sebastian Martinez

Section 1. Additional tables & figures

Table A1. Municipalities targeted by Salud Mesoamerica Initiative and number of community health teams per municipality

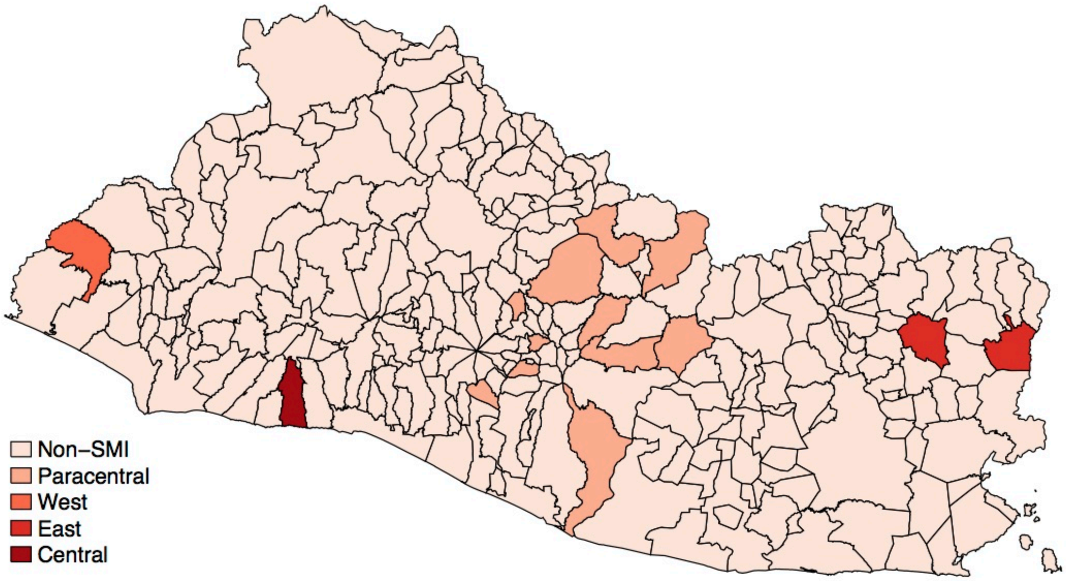

Geographical Region	SIBASI	Municipality	Community Health Teams in 2015 Rural	Urban	Total
Central	La Libertad	Chiltiupán	2	1	3
West	Ahuachapán	Tacuba	9	1	10
East	La Unión	El Sauce	2	1	3
East	Morazán	Sociedad	3	1	4
Paracentral	Cabañas	Ilobasco	12	1	13
Paracentral	Cabañas	Sensuntepeque	7	2	9
Paracentral	Cuscatlán	Monte San Juan	2	1	3
Paracentral	Cuscatlán	San Cristóbal	2	1	3
Paracentral	La Paz	San Antonio Masahuat	1	1	2
Paracentral	La Paz	Santa María Ostuma	2	1	3
Paracentral	San Vicente	Apasteque	6	1	7
Paracentral	San Vicente	San Esteban Catarina	1	1	2
Paracentral	San Vicente	San Ildefonso	2	1	3
Paracentral	San Vicente	Tecoluca	7	3	10
		**Total**	**58**	**17**	**75**

Notes: The map illustrates all the municipalities of El Salvador and highlights those municipalities targeted by SMI according to their geographical region. SIBASI (*Sistema Básico de Salud Integral*) is the administrative delimitation of the health system that has under its influence the primary and secondary care of a group of municipalities. SIBASIs are independent of each other, but they all depend from the central level. The number of community health teams is that reported by the MoH in 2015.

Figure A1. Sample performance report

Resultados del Monitoreo Externo del Desempeño

Periodo: Segundo Semestre del 2016

Ecos F:

Municipio:

Resumen de Resultados

Semestre	2015-II	2016-I	2016-II	2017-I	Puntos Posibles
	Puntos obtenidos				
**Planificación Familiar (PF)**					
1.Información sobre métodos de PF	0	5	5		5
2.Utilización métodos PF	15	15	15		15
**Control Prenatal**					
3. Control Prenatal Precoz	10	10	10		10
4. Control Prenatal con Calidad	10	10	10		10
**Parto y Puerperio**					
5. Partos Referidos por Ecos F	5	5	5		5
6. Parto Institucional	10	10	10		10
7. Control Puerperal Precoz	0	10	10		10
**Atención del Niño**					
8. Antiparasitarios	5	0	0		5
9. Micronutrientes	N.A.	15	15		15
10. SRO y Zinc para Diarrea	0	0	0		10
11. Vacuna SPR	N.A.	5	5		5
**Puntaje Total**	**55**	**85**	**85**		**100**
**Puntaje promedio ISM**	**42**	**69**	**73**		**100**

Interpretación

- De acuerdo a la evaluación externa la [redacted] obtuvo **85** puntos en el **segundo semestre del 2016** de un total de **100** posibles. El puntaje del Ecos F en este periodo es **mayor** que el promedio de puntos total de otros Ecos F de la Iniciativa Salud Mesoamérica (ISM) que es de **73** puntos.
- El Ecos F cumplió las metas establecidas en **9** de **11** indicadores, pero se puede mejorar en aquellos marcados en rojo.
- Se recomienda analizar en equipo el reporte con el detalle de cada indicador y los procesos clave para los servicios donde se logró cumplir la meta para mantener el nivel e identificar mejores prácticas y establecer compromisos de mejora en aquellos donde no se cumplió en conjunto con equipos supervisores del SIBASI, región y nivel central.
- *El equipo obtuvo el 85% de puntos posibles (85/100) en esta medición por lo que es acreedor al 85% del fondo de reconocimientos, es decir* ***850*** *dólares canjeables por bienes de la lista establecida.*

Notas

- Los resultados aquí presentados se basan en la Encuesta de la Prestación de Servicios de Salud para la Mujer y el Niño/a en Ecos Familiares realizada durante los meses de Septiembre a Noviembre del 2016 por un equipo externo contratado por la Iniciativa Salud Mesoamérica.
- Los resultados de la evaluación serán utilizados únicamente para los fines establecidos en la Iniciativa Salud Mesoamérica.
- El total de puntos posibles no incluye los indicadores donde hay pocas observaciones o no aplican en el periodo.
- P.O.=Se refiere a que hay muy pocas observaciones para establecer si se cumple la meta.
- N.A.= No aplica en ese periodo, porque se decidió cambiar la forma de medir el indicador para hacerlo más preciso.

Note: Sample report for a treatment team. Control reports were identical except for the highlighted part in the cover page that specifies the amount obtained.

Resultados del monitoreo externo del desempeño: Planificación Familiar

Periodo: Segundo Semestre del 2016

Ecos F:

Municipio:

1. Información sobre métodos de PF

Meta	Estatus	Puntaje Obtenido
**80%**	**Cumple meta**	**5/5**

- **Descripción** Este indicador mide el porcentaje de mujeres en edad fértil que cumplen criterios para recibir información de planificación familiar que la recibieron por parte del personal de salud.
- **Resultado** De las 11 mujeres en edad fértil que cumplen criterio para recibir información de PF visitadas al azar en el área de influencia del Ecos F, 9 recibieron información sobre métodos de PF modernos por personal de salud en los últimos seis meses.
- Con este resultado se concluye que se cumple la meta considerando el error de muestreo.
- **Recomendación** Se recomienda analizar en equipo el plan local.
- **Fuente** Encuesta de vivienda

2. Utilización de métodos de PF

Meta	Estatus	Puntaje Obtenido
**61%**	**Cumple meta**	**15/15**

- **Descripción** Este indicador mide el porcentaje de mujeres en edad fértil que cumplen criterios para planificación familiar que utilizan métodos modernos de PF.
- **Resultado** De las 19 mujeres en edad fértil que cumplen con criterio para planificar visitadas al azar en el área de influencia del Ecos F, 18 utilizaban métodos de planificación familiar modernos al momento de la visita.
- Con este resultado se concluye que se cumple la meta considerando el error de muestreo.
- **Recomendación** Se recomienda analizar en equipo el plan local.
- **Fuente** Encuesta de vivienda

Definiciones clave de Planificación Familiar

- **Mujeres que cumplen criterios de planificación familiar**. Para propósitos de la evaluación, éstas son aquellas mujeres en edad reproductiva (de 15 a 49 años de edad) excluyendo las siguientes:
  - mujeres en menopausia
  - con histerectomía
  - vírgenes
  - que no tienen relaciones sexuales y
  - aquellas embarazadas o tratando de quedar embarazadas.
- Las mujeres que cumplen criterios para recibir información de planificación familiar son las mismas que las anteriores pero excluye a las mujeres esterilizadas.
- *Recordar que las mujeres vírgenes o que no tienen relaciones sexuales no se deben dejar fuera de las acciones de promoción de planificación familiar ya que en cualquier momento pueden tener la necesidad de planificar.*
- *Es importante identificar a las mujeres que cumplen criterios para planificar o para recibir información de PF en el área de influencia del Ecos F para poder proveerles información adecuada y satisfacer su necesidades de planificación.*
- **Métodos modernos de planificación familiar :** Los métodos de planificación modernos incluyen:
  - esterilización femenina o masculina
  - dispositivo intra-uterino DIU
  - inyectables
  - implantes
  - píldoras anticonceptivas
  - píldora de emergencia
  - preservativo masculino o femenino
  - diafragma
  - esponja espemicida
- *Al recomendar un método de planificación familiar recuerde valorar a la paciente y conocer sus necesidades para poder identificar el método de planificación más adecuado para ella.*

Resultados del monitoreo externo del desempeño: Control Prenatal Parto y Puerperio

Periodo: Segundo Semestre del 2016

Ecos F: [redacted]
Municipio: [redacted]

3. Control Prenatal Precoz

Meta	Estatus	Puntaje Obtenido
**80%**	**Cumple meta**	**10/10**

- **Descripción** Este indicador mide el porcentaje de mujeres embarazadas cuyo primer control prenatal ocurrió antes de las 12 semanas de gestación según expediente.
- **Resultado** De los 7 expedientes de mujeres embarazadas seleccionados al azar en el Ecos F, 6 tuvieron su primer control prenatal antes de las 12 semanas de gestación.
- Con este resultado se concluye que se cumple la meta considerando el error de muestreo.
- **Recomendación** Se recomienda analizar en equipo el plan local.
- **Fuente** Encuesta en establecimientos

4. Control Prenatal con Calidad

Meta	Estatus	Puntaje Obtenido
**80%**	**Cumple meta**	**10/10**

- **Descripción** Este indicador mide el porcentaje de mujeres con parto en los últimos 4 meses (excluyendo el más reciente) que recibieron el control prenatal con calidad de acuerdo a las mejores prácticas en expedientes.
- **Resultado** De los 1 expedientes de mujeres con parto en el periodo evaluado seleccionados al azar en el Ecos F, 1 fue realizada de acuerdo a las mejores prácticas.
- Con este resultado se concluye que se cumple la meta considerando el error de muestreo.
- **Recomendación** Se recomienda analizar en equipo el plan local.
- **Fuente** Encuesta en establecimientos

Resultados por criterio de mejores prácticas en control prenatal

Criterio Clínico	% Cumple	Criterio Clínico[3/]	% cumple	Exámenes de...[4/]	% cumple
Al menos 4 APN	100%	FCF	100%	...Glucosa	100%
Peso[1/]	100%	Movimientos fetales	100%	...VIH	100%
Presión arterial[1/]	100%			...Hemoglobina	100%
Altura uterina[2/]	100%			...Orina	100%

Notas: Los porcentaje son respecto al total de expedientes revisados. Un expediente tiene que cumplir con todos los criterios para ser considerado como de mejores prácticas. Los criterios subrayados son aquellos que están por debajo del 80% [1/] Se mide en todas las visitas. [2/] Se mide a partir de las 14 semanas. [3/] Se mide a partir de las 20 semanas. [4/] Se mide que esté en el expediente al menos una vez.

5. Partos Referidos por Ecos F

Meta	Estatus	Puntaje Obtenido
**100%**	**Cumple meta**	**5/5**

- **Descripción** Este indicador mide el porcentaje de mujeres con parto en los últimos 4 meses (excluyendo el más reciente) que fueron referidas a una institución de salud como parte del plan de parto en expedientes.
- **Resultado** De los 1 expedientes de mujeres con parto en el periodo evaluado seleccionados al azar en el Ecos F, 1 tenía plan de parto y estaba establecida una institución médica como lugar de parto.
- Con este resultado se concluye que se cumple la meta considerando el error de muestreo.
- **Recomendación** Se recomienda analizar en equipo el plan local.
- **Fuente** Encuesta en establecimientos

6. Parto Institucional

Meta	Estatus	Puntaje Obtenido
**94%**	**Cumple meta**	**10/10**

- **Descripción** Este indicador mide el porcentaje de mujeres con parto en los últimos 4 meses (excluyendo el más reciente) que tenían registrado el parto institucional en expedientes.
- **Resultado** De los 1 expedientes de mujeres con parto en el periodo evaluado seleccionados al azar en el Ecos F, 1 tenía registrado el parto institucional ya sea con la copia del egreso, la hoja de control postparto o en algún otro lugar del expediente.
- Con este resultado se concluye que se cumple la meta considerando el error de muestreo.
- **Recomendación** Se recomienda analizar en equipo el plan local.
- **Fuente** Encuesta en establecimientos

7. Control Puerperal Precoz

Meta	Estatus	Puntaje Obtenido
**92%**	**Cumple meta**	**10/10**

- **Descripción** Este indicador mide el porcentaje de mujeres con parto en los últimos 4 meses (excluyendo el más reciente) que recibieron atención postnatal durante la semana posterior al parto según expediente.
- **Resultado** De los 1 expedientes de mujeres con parto en el periodo evaluado seleccionados al azar en el Ecos F, 1 tenía registrada la atención puerperal y esta fue realizada durante la semana posterior al parto.
- Con este resultado se concluye que se cumple la meta considerando el error de muestreo.
- **Recomendación** Se recomienda analizar en equipo el plan local.
- **Fuente** Encuesta en establecimientos

Resultados del monitoreo externo del desempeño: Atención del Niño

Periodo: Segundo Semestre del 2016

Ecos F: [redacted]
Municipio: [redacted]

8. Antiparasitarios

Meta	Estatus	Puntaje Obtenido
**66%**	**No cumple meta**	**0/5**

- **Descripción** Este indicador mide el porcentaje de niños de 12 a 59 meses que consumieron la dosis adecuada para la edad de antiparasitarios durante los últimos seis meses.
- **Resultado** De los 9 viviendas visitadas al azar con niños de 12 a 59 meses en el área de influencia del Ecos F, 2 consumieron la dosis adecuada para la edad de antiparasitarios en los últimos seis meses.
- Con este resultado se concluye que no se cumple la meta considerando el error de muestreo.
- **Recomendación** Se recomienda analizar en equipo el plan local.
- **Fuente** Encuesta de vivienda

9. Micronutrientes

Meta	Estatus	Puntaje Obtenido
**80%**	**Cumple meta**	**15/15**

- **Descripción** Este indicador mide el porcentaje de niños de 6 a 23 meses que tenían indicación de micronutrientes en expedientes y recibieron la dosis adecuada en farmacia.
- **Resultado** De los 16 expedientes de niños de 6 a 23 meses al momento de la visita seleccionados al azar en el Ecos F, 11 tenían indicación de micronutrientes registrada en el expediente y recibieron la dosis adecuada.
- Con este resultado se concluye que se cumple la meta considerando el error de muestreo.
- **Recomendación** Se recomienda analizar en equipo el plan local.
- **Fuente** Encuesta en establecimientos

10. SRO y Zinc para Diarrea

Meta	Estatus	Puntaje Obtenido
**50%**	**Cumple meta**	**10/10**

- **Descripción** Este indicador mide el porcentaje de madres de niños de 0 a 59 meses que mencionaron que tratarían a sus hijos con sales de rehidratación oral y zinc a sus hijos cuando tuvieran diarrea.
- **Resultado** De las 10 madres de niños de 0 a 59 meses visitadas al azar en el área de influencia del Ecos F, 5 madres mencionaron que tratarían a sus hijos con SRO y Zinc en caso de diarrea o trataron a sus hijos con SRO y Zinc si estos tuvieron un episodio de diarrea en las últimas dos semanas.
- Con este resultado se concluye que se cumple la meta considerando el error de muestreo.
- **Recomendación** Se recomienda analizar en equipo el plan local.
- **Fuente** Encuesta en vivienda

11. Vacuna SPR

Meta	Estatus	Puntaje Obtenido
**94%**	**Cumple meta**	**5/5**

- **Descripción** Este indicador mide el porcentaje de niños de 6 a 23 meses que tenían registro de la vacuna SPR según el libro de vacuna.
- **Resultado** De los 15 niños de 6 a 23 meses al momento de la visita seleccionados al azar en el Ecos F, 13 tenían registro de la vacuna SPR en el libro de vacuna.
- Con este resultado se concluye que se cumple la meta considerando el error de muestreo.
- **Recomendación** Se recomienda analizar en equipo el plan local.
- **Fuente** Encuesta en establecimientos

Figure A2. Timeline of data collection and key events

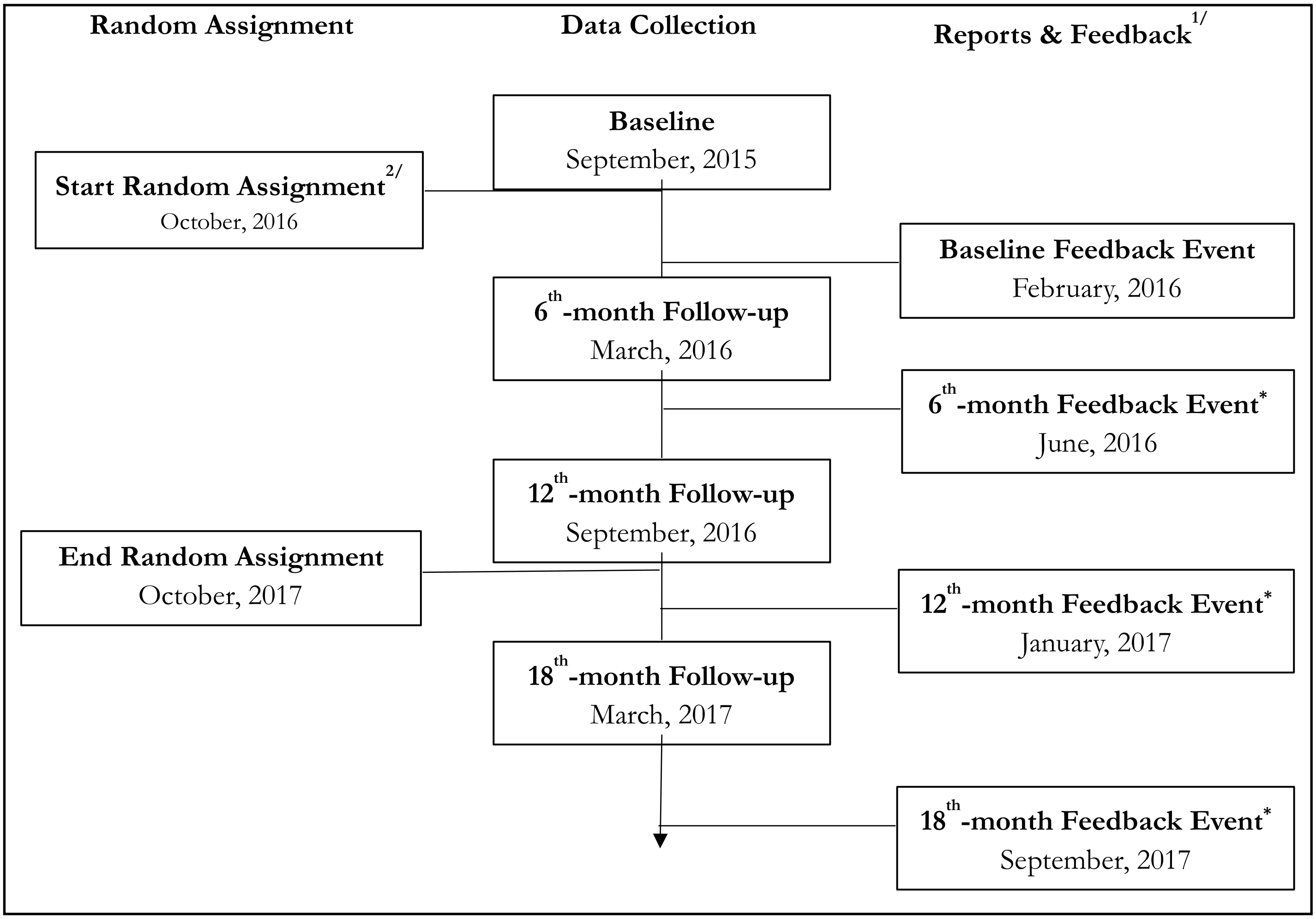

Notes: Dates of data collection refer to the start date. Each data collection lasted about two months and a half. The random assignment of teams into treatment and control ended after the 12th month follow-up was finalized.

[1/] Reports and feedback refer to the event in which teams received their performance report, the presentation of overall results as well as their performance certificates. The latter was only starting in the 6th month follow-up.

[2/] In the same date of the random assignment the incentive scheme was presented to teams.

* In these events, teams also received performance certificates.

Table A2. Definition of outcomes of interest and proxies

Category	Indicator and Definition	Source
Outreach	**Information on modern family planning***<br>Share of women 15 to 49 in need of contraception that received information on modern family planning methods by health personnel in the last six months.<br><br>Women in need of contraception are those age 15 to 49 excluding those sterilized, in menopause, that declare to be virgin or not sexually active, and those pregnant or trying to conceive.<br><br>Modern family planning methods include any of the following: injectable, contraceptive pills, female or male sterilization, intra uterine devices (IUD), implants, emergency contraception, female or male condoms, contraceptive diaphragm and sponges.	Household survey
Outreach	**Knowledge of treatment of diarrhea with ORS & Zinc***<br>Share of women 15 to 49 with children less than five with knowledge of treatment of diarrhea with oral rehydration salts and zinc at the time of the survey.<br><br>All women with these characteristics are asked in the survey about how they would treat their child if he/she felt ill with diarrhea and all options mentioned are recorded by enumerators. Enumerators do not read/provide any options in this question to women.	Household survey
Quality	**Quality prenatal care***<br>Share of women in the catchment area of community health teams with a delivery in the last four months (excluding the most recent month) that received prenatal care according to national clinical guidelines at the facility.<br><br>It is equal to one if during all prenatal care visits provided at the facility the women was assessed according to guidelines. The assessment guidelines include: for each visit regardless of gestational age measures of weight and blood pressure, for each visit after 13 weeks of gestation measurement of uterine height, for each visit after 19 weeks of gestation, fetal heart rate. In addition, women should have received blood tests assessing glucose, HIV and hemoglobin, and a urine test at least once during their prenatal care visits.	Medical Records
Quality	**Reference to institutional delivery***<br>Share of women in the catchment area of community health teams with a delivery in the last four months (excluding the most recent month) with reference to institutional delivery in the birth plan.	Medical Records
Timeliness	**Timely Prenatal Care**	
Timeliness	*Medical Record Proxy**<br>Share of women in the catchment area of community health teams that reached three months of gestation in the last six months for which the first prenatal care visit occurred prior to 12 weeks of gestation.<br><br>The gestational age at the time of the first visit is obtained by the difference between the date of the first prenatal care visit and the date of last menstrual cycle. If either piece of information is not found in the record it is considered that the woman did not received care, that is zero.<br>When compared to the household survey in Table 10 it is restricted to births occurring from October 2015 to October 2016, the comparable period with the survey.	Medical Records
Timeliness	*Household Survey*<br>Share of live births whose first prenatal care visit occurred prior to the first 12 weeks of gestation and was provided by a physician or a professional nurse.	Household Survey

Category	Indicator and Definition	Source
	When compared to the medical records proxy in Table 10 it is restricted to births occurring from October 2015 to October 2016, the comparable period with the medical records.	
Timeliness	**Timely Post-natal Care**	
Timeliness	*Medical Record Proxy** Share of women in the catchment area of community health teams with a delivery in the last four months (excluding the most recent month) that received postpartum care within a week from delivery by health personnel. The time of postpartum care is obtained by the difference between the dates of the first postnatal care visit for the woman and the date of delivery. If either piece of information is not found in the record it is considered that the woman did not received care, that is zero. When compared to the household survey in Table 10 it is restricted to births occurring from October 2015 to October 2016, the comparable period with the survey.	Medical Records
Timeliness	*Household Survey* Share of live births whose first postpartum care visit occurred within a week after delivery and was provided by either a physician, a nurse or a community health worker either at home or at a health facility. When compared to the medical records proxy in Table 10 it is restricted to births occurring from October 2015 to October 2016, the comparable period with the medical records.	Household Survey
Utilization	**Use of modern family planning methods*** Share of women 15 to 49 in need of contraception using a modern family planning method at the time of the survey <br>Women in need of contraception are those age 15 to 49 excluding those sterilized, in menopause, that declare to be virgin or not sexually active, and those pregnant or trying to conceive. <br>Modern family planning methods include any of the following: injectable, contraceptive pills, female or male sterilization, intra uterine devices (IUD), implants, emergency contraception, female or male condoms, contraceptive diaphragm and sponges.	Household survey
Utilization	**Institutional Delivery**	
Utilization	*Medical Record Proxy** Share of women in the catchment area of community health teams with a delivery in the last four months (excluding the most recent month) that delivered in a health facility according to the records. When compared to the household survey in Table 10 it is restricted to births occurring from October 2015 to October 2016, the comparable period with the survey.	Medical Records
Utilization	*Household Survey* Share of live births delivered in a health facility by skilled provider (physician or professional nurse). When compared to the medical records proxy in Table 10 it is restricted to births occurring from October 2015 to October 2016, the comparable period with the medical records.	Household survey
Utilization	**Micronutrients for children**	
Utilization	*Medical Record Proxy** Share of children age 6 to 23 months at the time of data collection in the catchment area of community health teams that were prescribed micronutrients sachets in the last six months according to the medical record.[1/]	Medical Records
Utilization	*Household Survey*	Household survey

Category	Indicator and Definition	Source
	Share of children age 6 to 23 months old that consumed 50 or more micronutrients sachets in the last six months according to maternal recall.	
Utilization	**Deworming pills consumption***<br>Share of children age 18 to 59 months old that consumed at least two deworming pills in the last six months according to maternal recall.	Household survey
Utilization	**Measles, Mumps and Rubella (MMR) Vaccination**	
Utilization	*Medical Record Proxy**<br>Share of children age 12 to 23 months old in the catchment area of community health teams immunized with the MMR vaccine according to the medical record. [2/]<br>When compared to the household survey in Table 10 it is restricted to children born from October 2014 to September 2015, the comparable period with the survey.	Medical Records
Utilization	*Household Survey*<br>Share of children age 12 to 23 months old that were immunized with the MMR vaccine according to their vaccination card.<br>When compared to the medical records in Table 10 it is restricted to children born from October 2014 to September 2015, the comparable period with the records.	Household survey
Non-contracted	**Diabetes detection**<br>Share of women 15 to 49 years old that were tested for diabetes in a health facility in the last six months.	Household survey
Non-contracted	**Hypertension Detection**<br>Share of women 15 to 49 years old that were tested for hypertension (blood pressure taken) in a health facility in the last six months.	Household survey
Non-contracted	**Cytology performed**<br>Share of women 15 to 49 years old that were tested for cancer in the uterine cervix (cytology or pap test) in a health facility in the last six months.	Household survey

Notes: *Indicator used to evaluate performance in the incentive scheme.

[1/] This definition was changed to micronutrients provided to children 6 to 23 months in the last six months after baseline since it was considered a better proxy for the incentive scheme. The definition in the table is the only one comparable across all rounds and hence the one used in the analysis.

2/ At baseline the source of verification for the incentive scheme was the clinical record of children but was changed to vaccination records after baseline since it was considered more comprehensive. The definition in the table is the only one comparable across all rounds and hence the one used in the analysis.

Table A3. Sample by source and verification cycle

	Baseline	6-month	12-month	18-month	Total	Average per cycle
**Panel A. Full Sample by Verification Cycle**						
Dwellings in Survey						
Total Surveyed	2,421	2426	2254	2130	9231	2308
With Eligible Women	1574	1673	1694	1519	6460	1615
Completed	1303	1523	1549	1413	5788	1447
With Children Less than 5	553	930	881	761	3125	781
With Children Less than 2	331	482	469	380	1662	416
Medical Records						
Pregnancies	794	884	761	786	3225	806
Deliveries	1127	1148	1152	1207	4634	1159
Children Age 6 to 23 months	1314	1364	1271	1393	5342	1336
**Panel B. Main Analysis Sample by Verification Cycle**						
Dwellings in Survey						
Total Surveyed	2,056	2,063	1,982	1,815	7,916	1979
With Eligible Women	1,343	1421	1510	1299	5,573	1393
Completed	1098	1,288	1,388	1,213	4987	1247
With Children Less than 5	464	775	798	663	2700	675
With Children Less than 2	280	395	421	341	1437	359
Medical Records						
Pregnancies	733	758	694	666	2851	713
Deliveries	1012	967	1019	1027	4025	1006
Children Age 6 to 23 months	1185	1167	1145	1189	4686	1172

Notes: Panel A presents a summary of the total sample collected in each verification cycle. Panel B restricts the sample to those 64 teams in which data on all outcomes for all 4 verification cycles were collected. The data on the household survey, are the number of dwelling with the specified characteristics. The data on records refer to the total number of medical records reviewed.

Table A4. Community teams by blocks, administrative regions and random assignment

Adminstrative Region (SIBASI)	Rural Teams				Urban Teams		Total		
	Block 1		Block 2		Block 3				
	C	*T*	*C*	*T*	*C*	*T*	*C*	*T*	*Total*
Ahuachapán	3	6			1		4	6	10
Cabañas			10	9	1	2	11	11	22
Cuscatlán			2	2	1	1	3	3	6
La Libertad	1	1				1	1	2	3
La Paz			1	2	1	1	2	3	5
La Unión			1	1		1	1	2	3
Morazán	2	1				1	2	2	4
San Vicente	9	7			4	2	13	9	22
**Total**	**15**	**15**	**14**	**14**	**8**	**9**	**37**	**38**	**75**

Notes: The number in each cell is the number of community health teams. Random assignment was done within each block. C=Experimental Control, T=Experimental Treatment.

Table A5. Balance test on catchment area and community health team characteristics

	Control Mean	Mean Difference (Treatment-Control)	Std. Error	P-Value	N
	(1)	(2)	(3)	(4)	(5)
**Panel A. Dwelling Characteristics**					
Floor of durable material	0.6774	0.0313	(0.052)	[0.551]	1,054
Ceiling of durable material	0.4307	0.0029	(0.077)	[0.970]	1,054
Wall of durable material	0.8226	0.0333	(0.048)	[0.487]	1,054
Electricity	0.7732	-0.0337	(0.040)	[0.401]	1,054
Phone	0.0882	0.0224	(0.017)	[0.197]	1,054
Toilet	0.2448	-0.0753*	(0.044)	[0.093]	1,054
Bono Comunidades Solidarias	0.2922	0.0006	(0.067)	[0.993]	1,054
Dwelling with Children Less Than 2	0.2619	0.0188	(0.027)	[0.485]	1,054
**Panel B. Women Characteristics**					
Age	30.3	-0.1279	(0.630)	[0.840]	1,095
Single	0.3403	-0.0444	(0.035)	[0.206]	1,052
No Health Insurance	0.9382	-0.0104	(0.017)	[0.549]	1,051
Elementary Education or Less	0.8184	0.0221	(0.031)	[0.475]	1,052
**Panel C. Team Characteristics**					
**Personnel**					
Physicians	1.23	-0.1042	(0.194)	[0.594]	64
Professional Nurses	0.98	0.0075	(0.088)	[0.933]	64
Auxiliary Nurses	1.16	-0.3484	(0.269)	[0.200]	64
Community Health Workers	2.97	-0.2079	(0.234)	[0.377]	63
Multi-purpose personnel	0.73	-0.0102	(0.114)	[0.929]	64
**Administrative Region (SIBASI)**					
Ahuachapán	0.1250	0.0416	(0.080)	[0.606]	64
Cabañas	0.2813	-0.0300	(0.087)	[0.730]	64
Cuscatlán	0.0938	0.0015	(0.073)	[0.984]	64
La Libertad	0.0469	0.0222	(0.052)	[0.671]	64
La Paz	0.0469	0.0323	(0.052)	[0.535]	64
La Unión	0.0469	0.0323	(0.052)	[0.535]	64
Morazán	0.0625	-0.0118	(0.061)	[0.847]	64
San Vicente	0.2969	-0.0881	(0.101)	[0.386]	64

Notes: Sample of analysis is that of 64 community health teams. Column (2) presents the difference between treatment and control groups controlling for blocks. Standard errors clustered at the team level are presented on Column (3). Durable materials include concrete, brick, adobe, concrete blocks, and tiles. Toilet refers to those connected to sewage or a septic tank. *Bono Comunidades Solidarias* is a conditional cash transfer program in El Salvador. Panel A and B present characteristics measured from household surveys at baseline. Panel C presents community health team characteristics at end line (no baseline data are available for these variables).
* $p < 0.10$, ** $p < 0.05$, *** $p < 0.01$.

Table A6. Relationship of treatment assignment and data availability

	Non-missing main outcomes	Non-missing outcomes for common indicators in medical records and household surveys	P-value of difference
	(1)	(2)	(3)
Treatment	0.0314	0.0141	
	(0.084)	(0.118)	
p-value	[0.710]	[0.905]	[0.880]
Control Mean	0.8378	0.4324	
N	75	75	

Notes: Column (1) presents the result of a regression of a dummy variable equal to one if the community health team had data on all outcomes all four rounds for the incentive scheme on data collection and zero otherwise against an experimental treatment assignment indicator and block effects. Column (2) presents a similar estimate, but the dependent variable is equal to one if the community health team had data on all outcomes used for performance measurement and with their equivalent in household surveys were available in all four round of data collection. Column (3) presents the p-value of the difference between the estimate of Column (1) and (2). Standard errors are clustered at the team level and are presented in parenthesis. P-values are included in square brackets.
* p < 0.10, ** p < 0.05, *** p < 0.01.

Table A7. Balance on baseline characteristics in different samples

	Sample	Control Mean	Mean Difference (Treatment-Control)	Std. Error	P-Value	N	Community Health Teams
	(1)	(2)	(3)	(4)	(5)	(6)	(7)
**Panel A. Dwelling Characteristics**							
Floor durable material	1	0.6832	0.0397	(0.045)	[0.384]	1,253	75
	2	0.6774	0.0313	(0.052)	[0.551]	1,054	64
	3	0.6318	0.0902	(0.087)	[0.307]	592	33
Ceiling durable material	1	0.4278	0.0236	(0.067)	[0.725]	1,253	75
	2	0.4307	0.0029	(0.077)	[0.970]	1,054	64
	3	0.4223	0.0858	(0.096)	[0.376]	592	33
Wall durable material	1	0.8220	0.0517	(0.041)	[0.208]	1,253	75
	2	0.8226	0.0333	(0.048)	[0.487]	1,054	64
	3	0.7770	0.0680	(0.077)	[0.386]	592	33
Electricity	1	0.7837	-0.0338	(0.035)	[0.337]	1,253	75
	2	0.7732	-0.0337	(0.040)	[0.401]	1,054	64
	3	0.7449	-0.0446	(0.053)	[0.405]	592	33
Phone	1	0.0958	0.0205	(0.016)	[0.214]	1,253	75
	2	0.0882	0.0224	(0.017)	[0.197]	1,054	64
	3	0.0878	0.0189	(0.022)	[0.397]	592	33
Toilet	1	0.2442	-0.0327	(0.040)	[0.417]	1,253	75
	2	0.2448	-0.0753*	(0.044)	[0.093]	1,054	64
	3	0.2078	-0.0576	(0.061)	[0.354]	592	33
Bono Comunidades Solidarias	1	0.3128	-0.0053	(0.058)	[0.927]	1,253	75
	2	0.2922	0.0006	(0.067)	[0.993]	1,054	64
	3	0.3108	-0.0937	(0.102)	[0.366]	592	33
Dwelling with Children Less Than 2	1	0.2586	0.0199	(0.024)	[0.405]	1,253	75
	2	0.2619	0.0188	(0.027)	[0.485]	1,054	64
	3	0.2905	0.0126	(0.039)	[0.748]	592	33
**Panel B. Women Characteristics**							
Age	1	30.1778	-0.1377	(0.596)	[0.818]	1,299	75
	2	30.2676	-0.1279	(0.630)	[0.840]	1,095	64
	3	30.3518	0.5379	(0.788)	[0.500]	614	33
Single	1	0.3445	-0.0462	(0.032)	[0.149]	1,251	75
	2	0.3403	-0.0444	(0.035)	[0.206]	1,052	64
	3	0.3176	-0.0450	(0.045)	[0.329]	592	33
No Health Insurance	1	0.9344	-0.0137	(0.017)	[0.418]	1,250	75
	2	0.9382	-0.0104	(0.017)	[0.549]	1,051	64
	3	0.9443	-0.0164	(0.025)	[0.523]	592	33
Elementary Education or Less	1	0.8145	0.0320	(0.028)	[0.250]	1,251	75

	Sample	Control Mean	Mean Difference (Treatment-Control)	Std. Error	P-Value	N	Community Health Teams
	(1)	(2)	(3)	(4)	(5)	(6)	(7)
	2	0.8184	0.0221	(0.031)	[0.475]	1,052	64
	3	0.8345	0.0120	(0.041)	[0.773]	592	33
**Panel C. Units Characteristics**							
**Personnel**							
Physicians	1	1.2267	-0.1068	(0.159)	[0.505]	75	75
	2	1.2344	-0.1042	(0.194)	[0.594]	64	64
	3	1.2727	-0.2724	(0.417)	[0.519]	33	33
Professional Nurses	1	0.9867	0.0220	(0.077)	[0.777]	75	75
	2	0.9844	0.0075	(0.088)	[0.933]	64	64
	3	0.9697	0.1417*	(0.081)	[0.088]	33	33
Auxiliary Nurses	1	1.1200	-0.3077	(0.217)	[0.160]	75	75
	2	1.1563	-0.3484	(0.269)	[0.200]	64	64
	3	1.2424	-0.6102	(0.629)	[0.340]	33	33
Community Health Workers	1	2.9054	-0.1559	(0.204)	[0.446]	74	74
	2	2.9683	-0.2079	(0.234)	[0.377]	63	63
	3	3.1250	-0.3370	(0.235)	[0.161]	32	32
Multi-purpose personnel	1	0.7467	-0.0173	(0.102)	[0.866]	75	75
	2	0.7344	-0.0102	(0.114)	[0.929]	64	64
	3	0.7273	0.1593	(0.153)	[0.305]	33	33
**Administrative Region (SIBASI)**							
Ahuachapán	1	0.1333	0.0518	(0.074)	[0.487]	75	75
	2	0.1250	0.0416	(0.080)	[0.606]	64	64
	3	0.1212	0.0201	(0.116)	[0.863]	33	33
Cabañas	1	0.2933	-0.0047	(0.080)	[0.953]	75	75
	2	0.2813	-0.0300	(0.087)	[0.730]	64	64
	3	0.3030	-0.2397*	(0.138)	[0.092]	33	33
Cuscatlán	1	0.0800	-0.0031	(0.063)	[0.960]	75	75
	2	0.0938	0.0015	(0.073)	[0.984]	64	64
	3	0.1515	0.0327	(0.141)	[0.818]	33	33
La Libertad	1	0.0400	0.0251	(0.045)	[0.579]	75	75
	2	0.0469	0.0222	(0.052)	[0.671]	64	64
	3	0.0606	-0.0277	(0.073)	[0.706]	33	33
La Paz	1	0.0667	0.0235	(0.058)	[0.685]	75	75
	2	0.0469	0.0323	(0.052)	[0.535]	64	64
	3	0.0000	0.0000	(0.000)	.	33	33
La Unión	1	0.0400	0.0251	(0.045)	[0.578]	75	75

	Sample	Control Mean	Mean Difference (Treatment-Control)	Std. Error	P-Value	N	Community Health Teams
	(1)	(2)	(3)	(4)	(5)	(6)	(7)
	2	0.0469	0.0323	(0.052)	[0.535]	64	64
	3	0.0606	0.1090	(0.082)	[0.192]	33	33
Morazán	1	0.0533	-0.0016	(0.052)	[0.976]	75	75
	2	0.0625	-0.0118	(0.061)	[0.847]	64	64
	3	0.0303	0.0704	(0.071)	[0.326]	33	33
San Vicente	1	0.2933	-0.1162	(0.092)	[0.209]	75	75
	2	0.2969	-0.0881	(0.101)	[0.386]	64	64
	3	0.2727	0.0352	(0.136)	[0.798]	33	33

Notes: Sample 1 refers to the full sample of 75 community health teams. Sample 2 refers to the subset of 64 teams with data on all outcomes for the performance score in all four rounds. Sample 3 refers to the subset of teams with data on all outcomes on all outcomes for the performance score in all four rounds and data on comparable indicators from the household survey. The third column presents the difference between treatment and control groups controlling for blocks. Standard errors clustered at the team level are presented on Column (4). Durable materials include concrete, brick, adobe, concrete blocks, and tiles. Toilet refers to those connected to sewage or a septic tank. *Bono Comunidades Solidarias* is a conditional cash transfer program in El Salvador. Panel A and B present characteristics measured from household surveys at baseline. Panel C presents community health team characteristics at end line (no baseline data are available for these variables).

* $p < 0.10$, ** $p < 0.05$, *** $p < 0.01$.

Table A8. Treatment Effect on Individual Indicators by Domain

	Control Mean	Baseline	Post-treatment (No controls)	Post-treatment (Controlling for Baseline)
	(1)	(2)	(3)	(4)
**Community Outreach**				
Information on modern family planning (HS)	0.5038	-0.0002	0.0567	0.0580*
		(0.046)	(0.035)	(0.035)
p-value		[0.996]	[0.109]	[0.098]
N		592	1,761	1,761
Knowledge of treatment of diarrhea with ORS & Zinc (HS)	0.0708	0.0185	0.0804**	0.0787**
		(0.030)	(0.037)	(0.035)
p-value		[0.540]	[0.033]	[0.027]
N		552	1,630	1,630
**Quality of Care**				
Quality prenatal care (MR)	0.5870	-0.1116*	0.0754**	0.0843**
		(0.057)	(0.035)	(0.033)
p-value		[0.057]	[0.034]	[0.013]
N		639	1,264	1,264
Reference to institutional delivery (MR)	0.8727	-0.0153	0.0212	0.0203
		(0.044)	(0.015)	(0.014)
p-value		[0.728]	[0.173]	[0.157]
N		638	1,264	1,264
**Timeliness of care**				
Timely Prenatal Care (MR)	0.7264	0.0276	0.0256	0.0187
		(0.036)	(0.028)	(0.025)
p-value		[0.452]	[0.360]	[0.462]
N		908	1,849	1,849
Timely Post-natal Care (MR)	0.5807	0.0638	0.0889**	0.0808**
		(0.069)	(0.040)	(0.039)
p-value		[0.359]	[0.032]	[0.042]
N		638	1,264	1,264
**Utilization**				
Use of modern family planning methods (HS)	0.7444	0.0096	0.0267	0.0340
		(0.048)	(0.030)	(0.025)
p-value		[0.843]	[0.380]	[0.184]
N		592	1,761	1,761
Use of modern family planning methods (HS)	0.7444	0.0096	0.0267	0.0340
		(0.048)	(0.030)	(0.025)
p-value		[0.843]	[0.380]	[0.184]
N		592	1,761	1,761

	Control Mean (1)	Baseline (2)	Post-treatment (No controls) (3)	Post-treatment (Controlling for Baseline) (4)
Institutional Delivery (MR)	0.7857	-0.0223	0.0486*	0.0476*
		(0.067)	(0.026)	(0.026)
p-value		[0.741]	[0.067]	[0.068]
N		638	1,264	1,264
Micronutrients for children (MR)	0.8791	-0.0239	0.0299	0.0320
		(0.036)	(0.027)	(0.027)
p-value		[0.512]	[0.279]	[0.240]
N		814	2,279	2,279
Deworming pills consumption (HS)	0.4084	0.0422	0.0535*	0.0450
		(0.056)	(0.032)	(0.031)
p-value		[0.455]	[0.098]	[0.148]
N		431	1,431	1,431
MMR Vaccination (MR)	0.6448	-0.0681	0.0372	0.0518
		(0.060)	(0.035)	(0.035)
p-value		[0.259]	[0.294]	[0.140]
N		814	2,279	2,279
**Non-contracted outcomes**				
Diabetes detection (HS)	0.1743	0.0083	0.0115	0.0106
		(0.031)	(0.017)	(0.017)
p-value		[0.792]	[0.495]	[0.525]
N		1,096	3,166	3,166
Hypertension Detection (HS)	0.4661	0.0756**	0.0324	0.0143
		(0.033)	(0.025)	(0.024)
p-value		[0.024]	[0.206]	[0.548]
N		1,098	3,170	3,170
Cytology performed (HS)	0.2789	0.0485	0.0087	0.0029
		(0.030)	(0.026)	(0.024)
p-value		[0.107]	[0.740]	[0.905]
N		1,097	3,174	3,174
Teams		64	64	64

Notes: The sample of analysis is of the 64 teams with data on all waves. HS in parenthesis denotes outcomes that are measured from the household survey. MR denotes outcomes measured from a medical record review. Column (1) presents the control mean at baseline. Column (2) presents the estimate of δ from baseline on the outcome of interest using equation (1), but excluding any baseline covariates. Column (3) presents the estimate of δ using the pooled 6th and 12th month follow-up data excluding baseline covariates. Column (4) presents the same estimate as (3) but includes as baseline covariate the mean team level outcome of interest at baseline. Standard errors are clustered at the team level and are presented in parenthesis. P-values are included in square brackets.
* p < 0.10, ** p < 0.05, *** p < 0.01.

Table A9. Comparison of ASTE by wave and domain

	Baseline	6-Month	12-months	18-months
	(1)	(2)	(3)	(4)
**ASTE of Contracted-Indicators by domain**				
ASTE Community Outreach	0.0358	0.0970	0.2570***	0.1128
	(0.069)	(0.088)	(0.083)	(0.097)
p-value	[0.603]	[0.271]	[0.002]	[0.244]
ASTE Quality of Care	-0.1361	0.1638***	0.1294*	0.1682***
	(0.095)	(0.061)	(0.071)	(0.064)
p-value	[0.152]	[0.007]	[0.067]	[0.009]
ASTE Timeliness of care	0.0954	0.0399	0.1664**	0.0720
	(0.089)	(0.064)	(0.066)	(0.066)
p-value	[0.286]	[0.536]	[0.012]	[0.275]
ASTE Utilization	-0.0323	0.0915**	0.0964*	0.0932*
	(0.065)	(0.046)	(0.049)	(0.048)
p-value	[0.620]	[0.046]	[0.051]	[0.052]
**ASTE Non-Contracted Outcomes**	0.0937*	-0.0359	0.0745**	0.0900*
	(0.053)	(0.044)	(0.037)	(0.054)
p-value	[0.079]	[0.415]	[0.045]	[0.096]
Community Health Teams		64	64	64

Notes: The sample of analysis is of the 64 teams with data on all waves. Column (1) presents the estimates of the average standardized treatment effect (ASTE) post-treatment controlling for team-level baseline outcomes by domain at baseline. Columns (2) to (4) presents the ASTE of teams at each follow-up. Standard errors are clustered at the team level and are presented in parenthesis. P-values are included in square brackets.
* p < 0.10, ** p < 0.05, *** p < 0.01.

Table A10. Comparison of ASTE using difference-in-difference and controlling for baseline

	Post-treatment (Controlling for Baseline)	DID	Difference (2) Vs (1)
	(1)	(2)	(3)
**ASTE of Contracted-Indicators by domain**			
ASTE Community Outreach	0.1748**	0.1769*	0.0021
	(0.069)	(0.100)	(0.059)
p-value	[0.011]	[0.078]	
ASTE Quality of Care	0.1427***	0.2436***	0.1010
	(0.054)	(0.093)	(0.089)
p-value	[0.008]	[0.009]	
ASTE Timeliness of care	0.1021*	0.0224	-0.0797
	(0.052)	(0.085)	(0.083)
p-value	[0.051]	[0.791]	
ASTE Utilization	0.0958**	0.1243*	0.0285
	(0.040)	(0.071)	(0.066)
p-value	[0.015]	[0.080]	
**ASTE Non-Contracted Indicators**	0.0215	-0.0520	-0.0735
	(0.031)	(0.059)	(0.048)
p-value	[0.485]	[0.377]	

Notes: The sample of analysis is of the 64 teams with data on all waves. Column (1) presents the estimates of the average standardized treatment effect (ASTE) post-treatment controlling for team-level baseline outcomes by domain, as presented on Panel A, Column (4) of Tables 6 through 9. Column (2) presents the difference-in-difference estimate with block effects of the pooled post-treatment follow-up at 6th and 12th months relative to baseline. Standard errors in Columns (1) and (2) are clustered at the team level and are presented in parenthesis. Column (3) presents the difference between the estimates of Column (2) and (1). Standard errors of the difference are obtained using bootstrap clustered at the team level. P-values are included in square brackets.
* p < 0.10, ** p < 0.05, *** p < 0.01.

Section 2. Robustness checks

A. Differential Attrition

We were unable to obtain complete data for 11 out of 75 teams (6 control and 5 treatment). The primary reason was security concerns in certain catchment areas due to increased gang violence. We test for differential attrition in two ways. First, we create a dummy variable equal to one if a team had data for all key outcomes[1] on all waves and zero otherwise and regress this against treatment assignment and block effects. Second, we, perform baseline balance tests on the analysis sub-sample of 64 teams with complete data, and the full sample of 75 teams. The results of both exercises are presented on Tables A6 and A7 of the first section in the Appendix. We find no evidence of systematic differences between treatment assignment and attrition as there are no statistically significant differences in the share of teams with data on all outcomes between treatment and control. Moreover, the baseline balance tests on the full sample are very similar to the analysis sample both in magnitude and statistical significance, suggesting that attrition was unrelated to treatment assignment.

B. Unobserved differences between teams

While the random assignment appears to have generated adequate baseline balance in covariates, as a robustness check we run a separate set of analysis using difference-in-differences (DID) which additionally controls for any time-invariant unobserved team characteristics. The downside of the DID specification is reduced precision. We test for differences between estimates from the DID specification and our preferred model presented in the results section using clustered bootstrap at the team level and stratified by block with 1000 replications to estimate the standard errors. The results, presented in Table A10 in the Appendix, indicate that in general there are no significant differences between both sets of estimates, and effect sizes are of a similar magnitude. Two exceptions are quality of care, in which the effect using DID is actually larger (0.24 Vs. 0.14 standard deviations) and timeliness of care in which the effect is substantially smaller (0.02 Vs. 0.10 standard deviations). However overall, the results from the DID analysis substantiate the results presented in section VI.

C. The effect of goods on productivity

The in-kind performance incentives consisted of goods that might affect productivity in subsequent rounds. For example, laptops could be used by teams to increase the efficiency of registration of key information or reduce the time to analyze information. If the in-kind incentives received in the first period affected productivity directly, this would confound the incentive effects in subsequent periods, as differences in productivity in the treatment group could be due to the additional goods obtained as well as incentives. While theoretically possible, we do not expect this bias to be large for two reasons. First, most goods selected by teams were used to increase comfort in the work place, including fans, microwaves, and water dispensers.[2] While these could affect work satisfaction, they are

---

[1] The key outcomes refer to those used in the main analysis and included in Tables 3 to 7 (11 performance outcomes and three non-contracted outcomes).

[2] The list of goods included air conditioners, refrigerators, microwaves, coffee makers, chairs, tables, laptops, printers, projectors, and digital cameras.

unlikely to have a direct effect on activities conducted in the field, such as community outreach or patient interactions required to improve performance indicators. Second, there were substantial delays in delivering the goods after the first six-month period since the acquisition process involved the purchase of small quantities of a diverse set of items using national procurement systems.

An indirect test of the potential effects of the goods on performance is given by the 6th month follow-up results presented in Table A9 in the Appendix, since no team had received any good before the first evaluation period. At the 6th month follow-up, the quality of care and utilization domains already exhibit statistically significant effects. The effect on quality is actually slightly larger at 6 months compared to the 12th months follow-up, and the effect on utilization remains at about the same magnitude in both rounds. The two domains for which there is no statistically significant effects at the 6th month follow-up but large increases at the 12th month follow-up are timeliness of care and community outreach. However, these are precisely the domains requiring effort on behalf of the health team in the field, where the goods offered as incentives are least likely to contribute directly to productivity.[3]

[3] For the case of community outreach, we speculate that the larger effect at the 12-month period might be explained by the fact that these activities are mostly performed by community health workers and the incentive scheme was presented first to representatives of teams which were mostly physicians and/or nurses. It might have been easier to mobilize these workers once the team achieved the first diploma and report with the recognition of their work and the amount received.
